# Virulence and Invasion Profiles of *Escherichia coli* Across One Health Reservoirs: Genomic Insights into High-Risk Clones and Their Defense Systems

**DOI:** 10.3390/pathogens15080812

**Published:** 2026-08-01

**Authors:** Sandra Martínez-Álvarez, Soraya Herrera-Espejo, Myriam Zarazaga, Ursula Höfle, María Eugenia Pachón-Ibáñez, Carmen Torres

**Affiliations:** 1 Area of Biochemistry and Molecular Biology, One Health-UR Research Group, University of La Rioja, 26006 Logroño, Spain; sandra.martinezal@alum.unirioja.es (S.M.-Á.); myriam.zarazaga@unirioja.es (M.Z.); 2 Clinical Unit of Infectious Diseases, Microbiology and Parasitology, Institute of Biomedicine of Seville (IBiS), Virgen del Rocio University Hospital/CSIC/University of Seville, 41013 Seville, Spain; 3 Health and Biotechnology (SaBio) Research Group, Institute for Game and Wildlife Research (CSIC-UCLM-JCCM), 13005 Ciudad Real, Spain; ursula.hofle@uclm.es; 4 CIBERINFEC, ISCIII—CIBER of Infectious Diseases, The Institute of Health Carlos III (ISCIII), 28029 Madrid, Spain

**Keywords:** *Escherichia coli*, CRISPR-Cas, antimicrobial resistance, virulence, pathogenesis, WGS, One Health

## Abstract

*Escherichia coli* is a genetically diverse species encompassing both commensal and pathogenic lineages capable of transitioning among various hosts. Within a One Health framework, we conducted a targeted screening of 38 *E. coli* strains isolated from wildlife, livestock, and food reservoirs to characterize their pathogenic potential by integrating genomic and phenotypic approaches. In vitro functional assays, including biofilm formation, surface motility, and adherence and invasion of HEK-293 epithelial cells, were statistically evaluated using the non-parametric Mann–Whitney U test. Phenotypic analyses revealed that extraintestinal pathogenic (ExPEC) and uropathogenic *E. coli* (UPEC) strains, particularly those belonging to the high-risk ST117 clone, exhibited significantly enhanced adherence and internalization capacities. These virulent phenotypes strongly correlated with specific genetic signatures involved in iron acquisition and epithelial invasion (*chuA*, *fyuA*, *vat*, and *tia*), underscoring that the convergence of ExPEC/UPEC determinants drives increased colonization potential. Genomic characterization further revealed that despite high virulence and widespread antimicrobial resistance, the CRISPR/Cas subtype I-E system was highly prevalent (93.8%), displaying structural variations frequently driven by insertion sequences. Spacer analyses identified limited homology to plasmids and phages, suggesting past mobilome interactions rather than active restriction of current horizontal gene transfer. Overall, these findings illustrate how phenotypic traits of high-risk clones match their genomic virulence platforms. The convergence of multidrug resistance and pathogenic fitness across human, animal, and environmental interfaces underscores the need for integrated molecular surveillance in a One Health context.

## 1. Introduction

*Escherichia coli* (Ec) is a highly adaptable bacterium that exists as a benign commensal in the intestines of humans and animals. However, certain strains have evolved into distinct pathogenic variants (pathotypes), each defined by a characteristic repertoire of virulence factors [1]. These pathotypes include Enteropathogenic *E. coli* (EPEC), Enterotoxigenic *E. coli* (ETEC), Shiga toxin-producing *E. coli* (STEC), and Enteroaggregative *E. coli* (EAEC) [1]. Additionally, extraintestinal pathogenic *E. coli* (ExPEC), encompassing uropathogenic *E. coli* (UPEC), are distinguished by their ability to cause diseases beyond the intestinal tract. All these pathotypes are primarily associated with infantile and traveler’s diarrhea, and in the case of EAEC, an enhanced ability to cause infections outside of the intestine (ExPEC), like causing a urinary tract infection (UTI) associated with the UPEC pathotype [1].

In this context, EPEC owes its pathogenicity primarily to the locus of enterocyte effacement (LEE) pathogenicity island, which encodes a type III secretion system (T3SS) [2]. Key virulence genes include *eae*, which encodes intimin. This adhesin facilitates intimate attachment to enterocytes, and *tir*, which encodes the translocated intimin receptor, is injected into host cells as a binding site for intimin [2]. Similarly, STEC is associated with severe conditions such as hemorrhagic colitis and hemolytic uremic syndrome due to the production of Shiga toxins (*stx1* and *stx2*) that inhibit protein synthesis in host cells [3]. Like EPEC, STEC possesses the LEE pathogenicity island, with the *tir* gene facilitating attachment to enterocytes [2,3]. For ETEC, its virulence is attributed to colonization factors and the production of enterotoxins such as heat-labile (*eltA*) and/or heat-stable (*sta*), which disrupt electrolyte homeostasis and lead to watery diarrhea [4]. In addition, some gene clusters (such as the colonization factor antigen, or *cfa*) facilitate adherence to the intestinal mucosa, playing a key role in the development of the infection [4].

ExPEC strains are by far the leading cause of UTIs (hence the label UPEC) and bacteremia [5,6]. According to these definitions, its pathogenicity are classified as follows: (i) as ExPEC if positive for two or more of the following genes *papAH* and/or *papC* (P fimbriae), *sfa-focDE* (S and F1C fimbriae), *afa-draBC* (Dr-binding adhesins), *iutA* (aerobactin siderophore system), and *kpsMII* (group 2 capsules), and as (ii) UPEC if positive for two or more of the following virulence coding genes, *chuA* (heme uptake), *fyuA* (yersiniabactin siderophore system), *vat* (vacuolating toxin), and *yfcV* (adhesin) [7,8].

The dissemination of pathogenic *E. coli* strains within the environment constitutes a significant public health challenge. Wildlife and production animals are critical reservoirs that facilitate the persistence and propagation of virulent strains as well as antibiotic resistance genes. The presence of extended-spectrum beta-lactamase (ESBL) genes confers resistance to a broad spectrum of beta-lactam antibiotics, which includes third-generation cephalosporins. These genes have been increasingly identified in *E. coli* from various sources, thereby playing a pivotal role in the antimicrobial challenges posed by the One Health perspective [9]. Consequently, advancements in genomic technologies have proven essential in clarifying the complexities associated with the pathogenesis and resistance mechanisms of *E. coli*. The Clustered Regularly Interspaced Short Palindromic Repeat (CRISPR)/Cas systems play a critical role in safeguarding bacteria against foreign genetic entities, including plasmids and bacteriophages [9]. Two phylogenetically distinct systems have been identified in *E. coli*: subtype I-E and subtype I-F1. The I-E system is composed of two CRISPR arrays (CRISPR-1 and CRISPR-2) and a set of eight associated *cas* genes that coordinate the acquisition and interference phases of adaptive immunity. In contrast, the I-F1 system includes six *cas* genes flanked by two additional CRISPR loci, CRISPR-3 and CRISPR-4. Functional differences between the two systems have been observed under laboratory conditions. While the I-E system appears to be transcriptionally silenced, primarily due to negative regulation by the global repressor H-NS, the I-F1 system is constitutively expressed. This constitutive activity enables subtype I-F1 to efficiently interfere with foreign genetic elements, such as phages and plasmids, thereby playing an active role in bacterial defense [9,10]. Diversity, evolution, and interactions among CRISPR-Cas systems within *E. coli*, as well as mobile genetic elements such as plasmids, significantly influence horizontal gene transfer. This interaction influences the acquisition and spread of antibiotic resistance genes relevant to bacterial evolution in wildlife and livestock, a matter that remains unclear [9,10]. Therefore, in a collection of 38 *E. coli* strains, 33 previously sequenced selected for their pathogenic potential [11,12,13,14,15,16], plus five new strains with relevant virulence factors, our aim is to comprehensively characterize the clinically significant serotypes detected in strains of animal, environmental, and food-derived products. For this purpose, a phylogenetic analysis was carried out to conduct a detailed examination of the genetic bases involved in the mobilization of virulence factors and clinical resistance genes, elements that had not previously been considered, and to relate these to in vitro assays examining functional phenotypic behaviors (in vitro adherence, invasion, biofilm formation, and motility).

## 2. Materials and Methods

### 2.1. Bacterial Collection

The final collection consisted of 38 *E. coli* strains selected from different One Health sources that had been surveyed in previous studies, to evaluate their pathogenic potential (Table S1) [12,13,14,15,16]. Strains were included based on the presence of specific genetic markers defining extraintestinal or diarrheagenic pathotypes (ExPEC, UPEC, EPEC, ETEC, and STEC). Following this criterion, 33 ESBL-producing *E. coli* isolates, previously sequenced (WGS), were pulled from the surveillance studies. These originated from white stork feces and nestling cloacal swabs (n = 23) [14,15], poultry farm manure and air (n = 3) [11,16], pig nasal samples (n = 5) [12,16], and retail chicken meat (n = 2) [13]. Additionally, five non-ESBL *E. coli* strains were added to the study: two from pigs (nasal and manure), two from chicken meat, and one from a stork nestling. These five strains had been left out of the original resistance surveys because they were phenotypic non-ESBLs, but they were included here because they carried the same core virulence factors as the pathotypes mentioned above, providing a non-resistant background for comparative purposes (Table S1).

### 2.2. Sequencing of the E. coli Genomes

Genomic DNA from *E. coli* strains was extracted using the QIAmp DNA Mini Kit (Qiagen, Hilden, Germany). Subsequently, libraries were prepared with the TruSeq DNA PCR-free Sample Preparation Kit (Illumina, San Diego, CA, USA), and 150 bp paired-end reads were sequenced in a NovaSeq 6000 System (Health in Code Facility). Illumina reads were quality-checked and trimmed using Trimmomatic v0.39 [17] to optimize their quality.

### 2.3. Genome Analysis

After filtering out low-quality reads, clean reads were submitted to the PLACNETw plasmid reconstruction tool [18] for whole-genome assembly. Manual pruning, combined with reference sequence comparisons, enabled clustering and the download of the core genome (chromosome) and accessory genome (plasmids) into separate files. PLACNETw assigned plasmids by identifying relaxases and replication initiator proteins associated with incompatibility groups (Inc). Multi-locus sequence typing profiles were made using the Achtman scheme through EnteroBase (http://enterobase.warwick.ac.uk) (accessed on 21 September 2024). AMR and virulence gene content analyses were done with ResFinder v4. 6 and VirulenceFinder v2. 0, respectively, by using 90% identity and minimum length thresholds [19,20,21]. The analysis of the presence of CRISPR/Cas systems and the number of spacer sequences was performed using CRISPRCasFinder v2.0.3 [22,23]. A BLASTn search for homologues of spacers was performed against the nucleotide collection (nr/nt) database with query coverage and identity >90%. As needed, if contigs were manually assembled, we also curated annotations and performed manual annotations using additional bioinformatic tools such as Artemis and SnapGene and relied on TnCentral (https://tncentral.ncc.unesp.br/blast/; accessed on 20 December 2024) (visual comparisons were performed with EasyFig v2.2.2 [24]). Phylogenetic analysis of the *cas* and *iss* genes were conducted with MEGA11 software [25], based on alignments generated through CLUSTALW and constructed with UPGMA.

In the phylogenomic study, the core genome was defined using Roary v3.13.0 with a protein BLAST identity of 80% and a core definition of 90% [26]. Subsequently, recombination was removed with Gubbins v3.2.1. Snp-dists v0.8.2 (https://github.com/tseemann/snp-dists) was used to extract the number of SNPs between all strains from the recombination-free core genome alignments. Finally, RAxML v8.2.12 was employed to construct the core genome phylogenetic tree, utilizing 100 replicates for bootstrap determination [27]. The resulting tree was visualized using iTol v5.5.1 (http://itol.embl.de/itol.cgi).

### 2.4. In Vitro Virulence Study

All assays were conducted in triplicate on different days, with two technical replicates each.

#### 2.4.1. Human Kidney Epithelial Cell Cultures and Infection

The human kidney epithelial cell line HEK-293 (ATCC CRL321) was maintained in culture in Dulbecco’s Modified Eagle Medium (DMEM, GE Healthcare LifeSciences, Salt Lake City, UT, USA), supplemented with 10% heat-inactivated fetal bovine serum (FBS, Gibco, Thermo Fisher Scientific, Madrid, Spain), 1% HEPES (Merck, Madrid, Spain), 50 μg/mL vancomycin (Normon Laboratories, Madrid, Spain), 20 μg/mL gentamicin (Normon Laboratories, Madrid, Spain), and 0.25 μg/mL amphotericin B (Gibco, Thermo Fisher Scientific, Madrid, Spain), in a humidified  incubator (37 °C, 5%CO_2_). The HEK-293 cell line underwent routinary passes every 3–4 days, with cells being seeded at a density of 10^5^ cells per well for 24 h in 24-well plates.

#### 2.4.2. Bacterial Adherence and Invasion of Cultured Human Kidney Epithelial Cells

Bacterial adherence and invasion assays were conducted in accordance with the previously established protocols [28]. Prior to infection, HEK-293 cells were washed twice with PBS. Concurrently, *E. coli* strains that had been cultured overnight were centrifuged (15 min at 4500 *g*) and subsequently resuspended in DMEM. Following this, the HEK-293 cells were incubated at a 1:1000 dilution (approximately 6.5 log_10_ CFU/mL) for 2 h of the overnight culture, which was confirmed in each experiment by viable colony counting after plating serial dilutions on blood agar plates. After incubation, for bacterial adherence assays, HEK-293 cells were rinsed five times with PBS; 200 μL of trypsin-EDTA (Gibco, Thermo Fisher Scientific, Madrid, Spain) was added and incubated for 10 min at 37 °C. Subsequently, 200 μL of 0.5% Triton X-100 (Merck) was incorporated, and the incubation was extended for an additional 2 min.

Regarding the bacterial invasion of HEK-293 cells, the MIC of gentamicin was determined for all strains to confirm susceptibility and to select a sufficient antibiotic concentration for extracellular killing. After 2 h of incubation, wells were washed twice with PBS, and then 256 μg/mL of gentamicin (Normon Laboratories) was added and incubated for one hour. This concentration of gentamicin ensured that extracellular bacteria were eliminated, as the isolates had MICs for gentamicin below this concentration. Then, 200 μL of trypsin-EDTA (Gibco, Thermo Fisher Scientific, Madrid, Spain) was added and incubated for 10 min at 37 °C. Later, 200 μL of 0.5% Triton X-100 (Merck, Madrid, Spain) was incorporated, and the incubation was extended for an additional 2 min. In all instances of bacterial adherence to or invasion of the HEK-293 cell line, serial dilutions of the Triton X-100 lysates were plated on blood agar, incubated overnight at 37 °C, and viable bacteria were enumerated by colony counting to determine CFU/mL.

#### 2.4.3. Biofilm Assay

The production of biofilm was quantified utilizing a method previously outlined in the literature [29]. Strains were cultured in 10 mL Luria–Bertani (LB, Merck) broth overnight at 37 °C and subsequently diluted to a concentration of 10^5^ CFU/mL in MHB. An aliquot of 200 µL of the cell suspension was dispensed into each well of a flat-bottom 96-well plate and incubated overnight at 37 °C. *E. coli* ATCC 25922 and *K. pneumoniae* CCT 9027 were included as positive controls for biofilm production, while sterile MHB served as the negative control. Each well was washed twice to eliminate non-adherent bacterial cells, after which 200 µL of 0.4% crystal violet dye (Merck, Madrid, Spain) was added. Following a 10-min incubation period, the wells were washed twice again, and 200 µL of 96% ethanol was added. A 15-min incubation ensued prior to the quantification of biofilm formation through the measurement of optical density (OD) at 580 nm, utilizing an Asys Microplate Reader. Biofilm production was normalized by setting the OD of the negative control and the reference strain as 0% and 100%, respectively.

#### 2.4.4. Surface Motility Assay

Surface motility was measured based on a previously described method [30]. Overnight cultures of each strain were adjusted to an optic density of 600 nm of 0.6 in PBS (Lonza, Basel, Switzerland). Then, 3 µL of the bacterial suspension was placed in the center of an LB plate containing 0.3% agarose. Plates were incubated at 37 °C with 80% humidity, and the radius of surface extension was measured at 24 h of incubation [30].

### 2.5. Statistical Analysis

Variations in bacterial adherence, invasion, biofilm formation, and motility were assessed using the Mann–Whitney U test, as provided by the software package SPSS (Version 26.0; Armonk, NY, USA: IBM Corp). The graphical representations were produced using GraphPad Prism 10. All *p*-values less than 0.05 were regarded as statistically significant.

### 2.6. Nucleotide Sequence Accession Numbers

The genome assemblies reported in this paper were deposited under NCB BioProject PRJNA1276316 for the five newly sequenced genomes and under PRJNA1066735, PRJNA954768, PRJNA1102028, PRJNA1149322, and PRJNA1077871.

## 3. Results

The 38 genomes of *E. coli* strains recovered from distinct sources (Table 1 and Table S1) were analyzed, focusing on the virulome (Section 3.1), associated plasmidome and resistome (Section 3.2), phylogenomics (Section 3.3), and their CRISPR/Cas systems (Section 3.4).

### 3.1. Virulome

**Figure 1:**
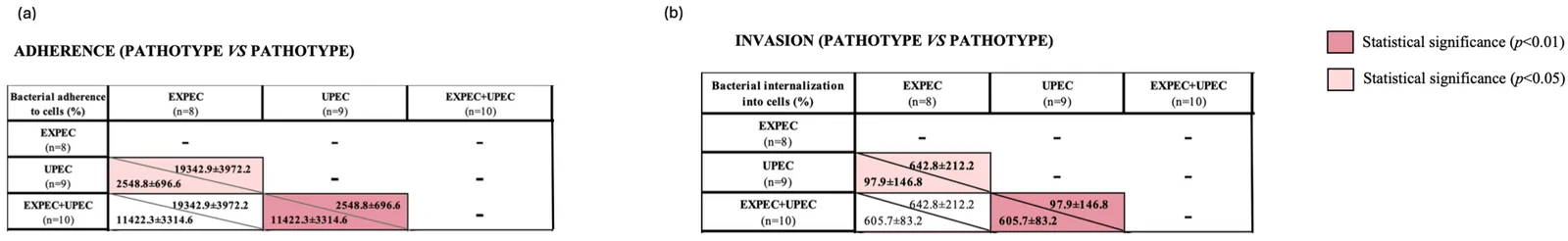
Mann–Whitney U analysis of the 38 *Escherichia coli* isolates grouped by pathotype for the interpretation of (**a**) adherence and (**b**) invasion to epithelial kidney cells (HEK-293 line).

We identified 47 virulence-related genes, of which 13 were primarily associated with relevant pathotypes. Within the extraintestinal group, we observed the pathotypes ExPEC (n = 8; *pap*, *sfa/focDE*, *afa/draBC*, *kpsMII*, *iutA*), UPEC (n = 9; *chuA*, *fyuA*, *vat or yfcV*), and a combination of ExPEC and UPEC (n = 10; characterized by a minimum of two of the previously described genes) (Table 1). In vitro adherence and invasion assays demonstrated that the pathotypes exhibiting the highest colonization rates corresponded to strains carrying genes associated with ExPEC and UPEC (Figure 1). Furthermore, significant differences among pathotypes were observed in both adherence and invasion assays (Figure 1)**.** Comparisons involving underrepresented pathotypes should be interpreted with caution. Statistical analyses were restricted to pathotype groups with adequate sample sizes (n ≥ 5). Groups with fewer than five isolates are shown for descriptive purposes only, and no statistical comparisons or inferential conclusions are presented for these groups.

Additionally, Figure S1 presents the statistical analyses conducted among groups with adequate sample sizes: (a) strain-by-strain comparisons, (b,c) ESBL type versus pathotype and vice versa, and (d,e) genetic lineage versus pathotype and vice versa. Due to the high prevalence of ExPEC and UPEC isolates and the potential chromosomal localization of the encoding genes in these pathotypes, we further compared the results according to their genetic lineages. Significant differences in cellular invasion were observed between the ExPEC and UPEC pathotypes within the high-risk clones ST117 and ST131, as well as between UPEC isolates belonging to ST117 and ST155 (Figure S1d). Although all ST117 isolates belonged to the same genetic lineage, the UPEC subgroup exhibited a significantly greater capacity to internalize into host cells than the ExPEC subgroup (Figure S1e). These findings suggest that ST117 UPEC isolates may harbor additional virulence determinants that enhance epithelial invasion and contribute to their pathogenicity in urinary tract infections.

**Figure 2:**
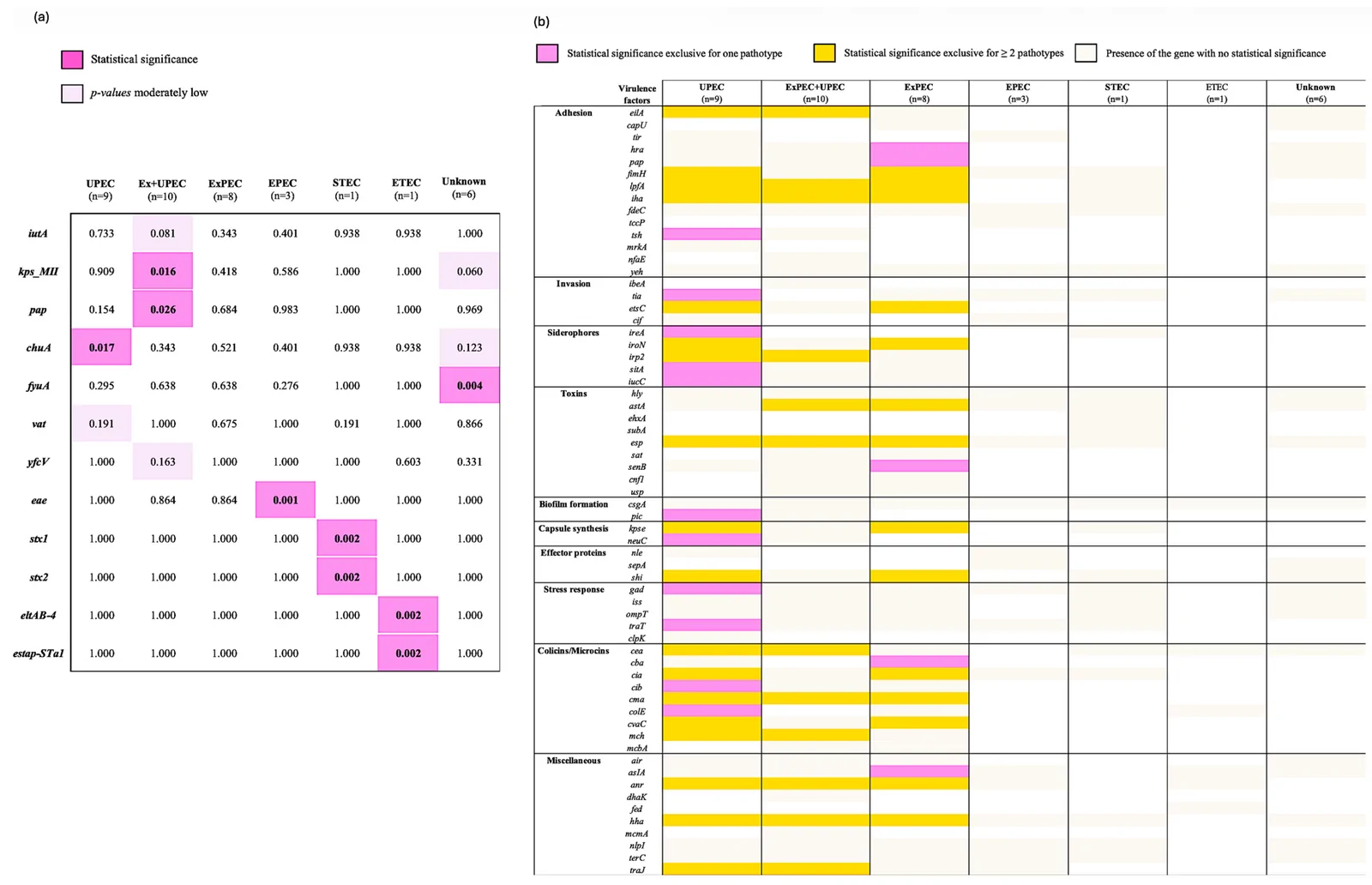
(**a**) Statistical analysis of the distribution within the virulence profiles encoding the different pathotypes detected in our study. (**b**) Overview of the additional virulence determinants encountered among pathogenic *E. coli* and their correlation with the different pathotypes analyzed. Exclusivity is denoted in pink, while genes detected across multiple pathotypes are highlighted in yellow.

We conducted a genomic analysis to correlate In vitro adherence and invasion with specific virulence factors. Our approach involved: (i) identifying which genes associated with each pathotype were evidently linked based on our findings, and (ii) investigating statistically significant relationships between virulence genes and bacterial pathotypes, particularly emphasizing those genes that displayed an exclusive correlation with a single pathotype. The results of this study are represented in a heat map, which illustrates the relationships between pathotypes and genes (Figure 2). It was first noted that the *chuA* gene showed a strong association with the UPEC pathotype. Additionally, the *kpsMII* and *pap* genes were linked to both ExPEC and UPEC and the *eae* gene was associated with EPEC, while the *stx1/2* genes corresponded to STEC, and the toxins *eltAB-4* and *stap-STa1* were related to ETEC, as previously described (Figure 2a). Regarding the complementary virulence factors identified, it has been observed that the ExPEC pathotype exhibited a notable specificity for the *hra* gene. In contrast, UPEC demonstrated specificity for the *tsh* gene, both of which play significant roles in cell adhesion. Additionally, the *tia* gene in UPEC is implicated in cell invasion (Figure 2b). The lack of significant associations for certain pathotypes such as EPEC, STEC, and ETEC in this analysis may primarily be attributed to the very limited sample sizes, with totals of only three, one, and one, respectively. Furthermore, the low specificity of the genes examined within these pathotypes may have contributed to this outcome. However, it is important to note that the presence of a given gene pool does not inherently guarantee its transcriptional activation or translational execution under experimental conditions. Consequently, these genomic profiles are presented as markers that align with higher pathogenic trends, providing a baseline for future functional evaluation.

### 3.2. Plasmidome and Resistome

PLACNETw reconstructions have indicated the presence of plasmids in all 38 genomes of *E. coli* strains studied, demonstrating a wide diversity in terms of size, replicon families, and the quantity per isolate, which ranges from one to five.

Table 2 summarizes the sizes and genetic characteristics of the small plasmids identified in 47.4% of the strains. In most instances, these plasmids contained only genes associated with replication and/or plasmid mobility. Nevertheless, they exhibited significant phylogenetic diversity, encoding replication proteins and relaxases that belong to four distinct families. Among the small mobilizable plasmids, an association was noted between the relaxase subfamily MOB_Q12_ and a Rep similar to the one implicated in pColE2 replication [col(156)]. Furthermore, due to its rarity among *Enterobacteriaceae*, we emphasize the detection of a 2376 bp plasmid (plasmid from X3078), which exhibits conserved domains of *orf2* and a replication protein (*repL*). In addition to the small mobilizable plasmids, several mini-plasmids were identified that contain minimal information for self-perpetuation (plasmids X7553). These mini-plasmids represent the shortest lengths within the entire collection, measuring between 1.5 and 2.2 kb, and exhibit homology to the pKST21 family [col(MG828)], which is classified as a mosaic group of plasmids that are exclusively transferable through vertical inheritance (*repA-repB*). Regarding the adaptive and cargo genes of the small plasmids, the majority appeared to be cryptic, as their detectable open reading frames (ORFs), ranging from one to three, encoded unknown hypothetical proteins. Notably, only three small plasmids were associated with colicin production, explicitly containing the *colE1*, *colE9*, and *colE10* operons. Furthermore, when conducting a comparative analysis of our set of plasmids against those cataloged in public databases, we found that 15 of them were either identical or highly similar (≥99%) to others previously documented among *Enterobacteriaceae* from human and livestock sources (Table 2).

The *iss* virulence factor, often linked to colibacillosis in avian species and observed in pColV/BM plasmids, was identified in 84.2% of the *E. coli* strains analyzed. As illustrated in Figure S2, the *iss* sequence correlated with elevated serum survival was found to cluster with the *iss* type 1 group, while a separate sequence characterized by lower survival, referred to as ‘type 13,’ was identified as a subcluster within the *iss* type 2 group. While all *iss* alleles were detected among our *E. coli*, the majority encoded the type 2 chromosomal structure.

**Figure 3:**
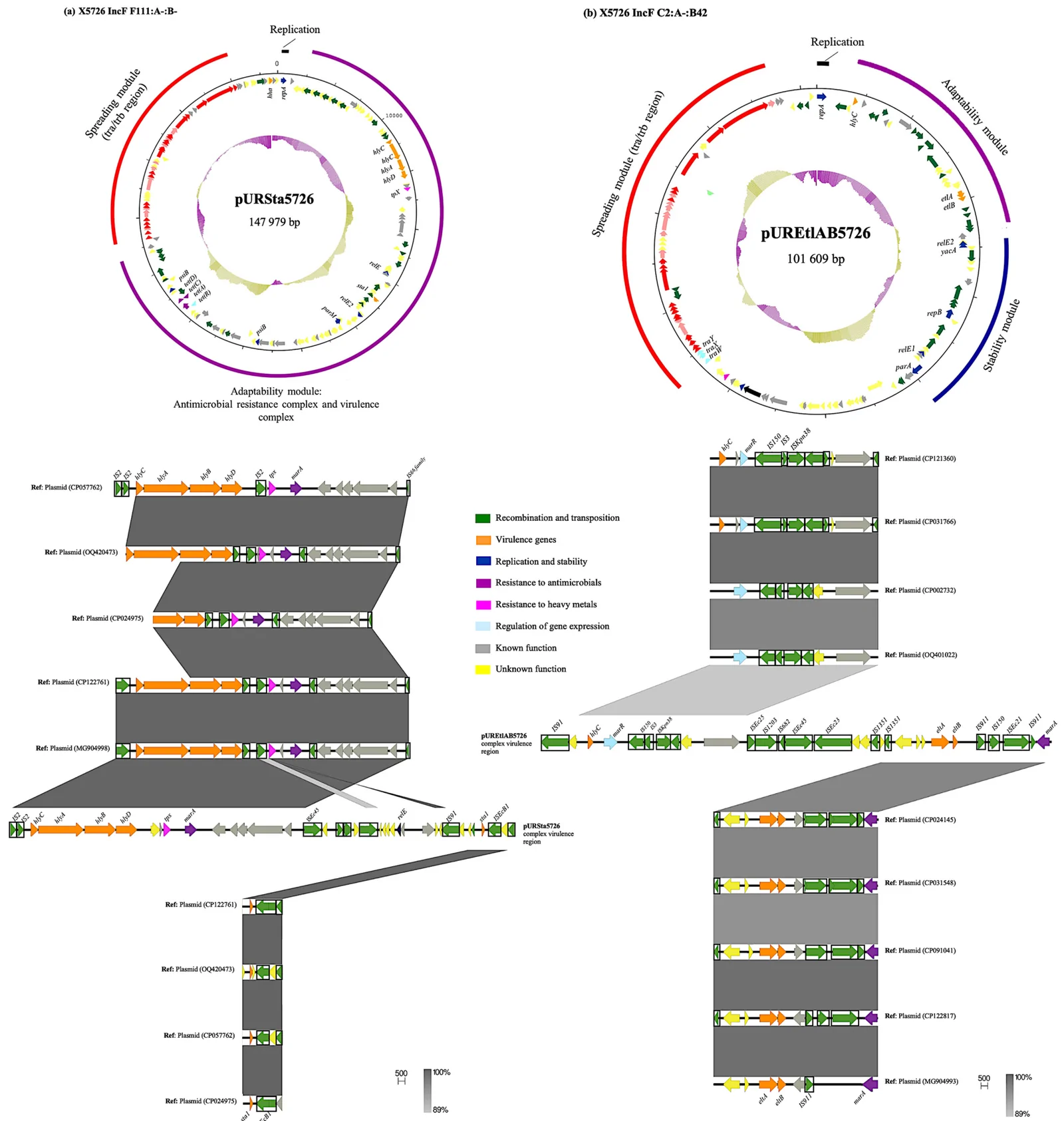
Circular maps of plasmids (**a**) pURSta5726 and (**b**) pUR5726eltAB. Linear illustration of the genetic environment of the virulence genes. The reference genomes used for BLASTn comparisons are indicated as “Ref. Genetic Location–Strain name–(accession number)”. (**a**) Second inner ring shows the fragments of truncated genes, and the forward and reverse coding sequences are shown in the third and fourth inner rings, respectively. They are shown as arrows (the direction of transcription is indicated by the arrowheads) and are colored according to their function, as shown in the legend. Names of functional regions are shown in the outer ring. The first inner ring shows a plot of the GC skew (yellow, above average; purple, below average). Coding reading frames are shown as arrows (the direction of transcription is indicated by the arrowheads) and are colored as described in (**a**). ISs are presented as boxes and the arrows within the boxes indicate the transposition genes. Vertical lines represent the IRs of ISs.

Upon examination of the larger plasmids, it was observed that the genes encoding the two toxins associated with the ETEC pathotype (*stA*, *eltAB*) were situated on IncF plasmids (F1: A-:B- for *stA*; C2: A-:B42 for *eltAB*), as depicted in Figure 3a,b. On the one hand, the pURSta5726 plasmid harboring the *stA1* toxk presented great length (147,979 bp), with the adaptability module containing various virulence and metal resistance genes (*tpx*, thioredoxin peroxidase), as well as several genetic platforms (Figure 3a). Firstly, the virulence factors *hlyABCD* are in a composite transposon flanked by IS*2*. Upstream are the *tpx* gene and the multidrug-resistance gene *marA*, presenting 100% homology with other identical structures detected in other plasmids previously described (Figure 3a). Downstream, there is the genetic platform IS*Ec43*-hp-IS*2*-IS*91*-hpos-*relE*-*cps* and subsequently the *stA1* toxin, which is flanked by IS*91* and IS*Ec43*, showing 100% homology with structures previously described in other plasmids (Figure 3a).

On the other hand, the pUREtlAB5726 plasmid carrying the *eltA* and *eltB* toxins was slightly smaller in size, with 101,609 bp (Figure 3b). The adaptability module consisted of a first region containing the virulence factor *hlyC* and the *marR* repressor of the multiple antibiotic resistance (*mar*) system, flanked by the insertion sequence IS*91* and the genetic platform IS*150*-IS*3*-IS*Kpn38*, with 89% homology. Upstream were the toxins *eltA* and *eltB* which were flanked by the genetic structures (i) IS*Ec25*-IS*1203*-IS*682*-IS*Ec45*-IS*Ec23*-IS*1351* and (ii) IS*911*-IS*150*-IS*Ec21*-IS*911*, together with the *marA* gene, which showed 100% homology with structures previously described in other plasmids (Figure 3b).

The 33 *E. coli* strains analyzed in previous studies exhibited numerous genetic determinants of clinical significance (*bla*_ESBL_ genes) that have been documented in prior research [11,12,13,14,15,16]. In addition to these genes, it is essential to emphasize the diversity of genetic platforms identified for mobilizing AMR determinants. As delineated in Table 1, among the extensive conjugative plasmids present, those in *E. coli* strains of the MOB_F12_ subfamily (IncF complex) predominated, succeeded by MOB_P12_ (predominantly IncI1). Despite a high resistance rate among this collection of *E. coli* from wildlife (21.0%), plasmids harboring AMR genes were categorized into various MOB/Inc families (MOB_F12_/IncF, MOB_P12_/IncI1, MOB_F11_/IncN, MOB_P3_/IncX1, and -/IncR). Among the four *bla*_TEM-1_ genes detected, four distinct genetic environments were observable. The most prevalent *bla*_TEM-1b_ variant was located upstream of the *tnpR* gene in Tn*1331* and was also flanked by two IS*91* elements. The *bla*_TEM-234_ variant, differing by three nucleotides, was identified within a composite IS*15*-*bla*_TEM-234_-IS*1*-IS*150*-IS*15* transposon, in which IS*1* and IS*150* elements were inserted downstream, likely recruiting this set of genes and integrating it into a larger complex via homologous recombination, as no direct duplications at the target site were observed.

**Figure 4:**
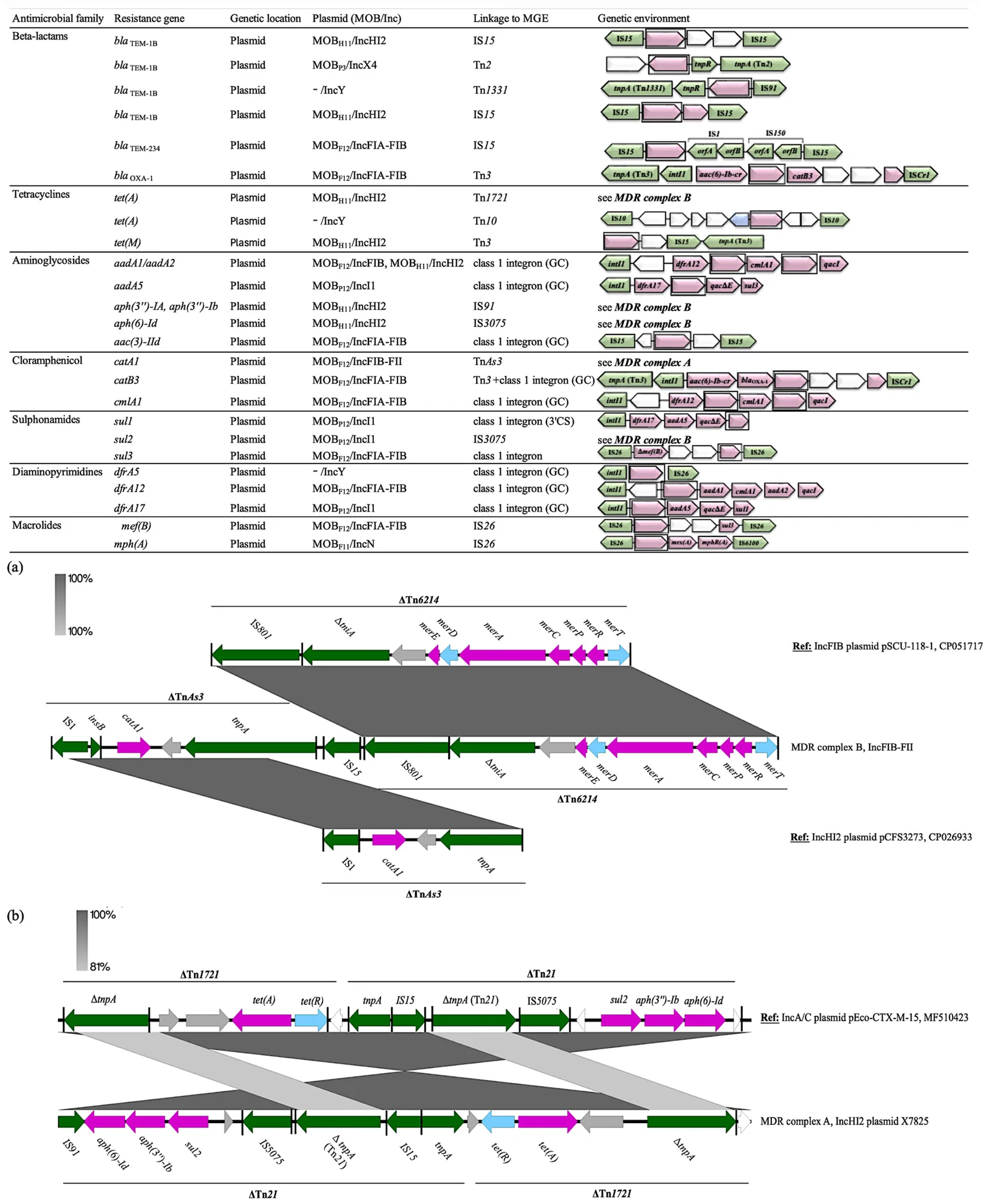
Genetic landscape and location of antimicrobial resistance determinants identified in our collection of *E. coli* from diverse origins. (**a**) BLAST comparison of a hybrid transposon Tn*21*-*1721* carrying an atypical class 1 integron and *tet*(A) in IncFIB-IncFII plasmids (MDR A complex); (**b**) BLAST comparison of the novel multi-resistance complex (MDR B complex) with other reference sequences from public databases. Genes are colored according to function: purple, drug or metal resistance; green, recombination/transposition; blue, replication and regulation of gene expression; gray, known function; white, unknown function.

Consequently, the previously uncharacterized ∆Tn*As3*-∆Tn*6214* complex emerged from multiple insertion events within the original ∆Tn*As3* backbone (Figure 4a). The genes encoding tetracycline efflux pumps, specifically *tet*(A) and *tet*(B), were located within the well-documented transposons Tn*1721* and Tn*10*, respectively. Incorporated within the commonly detected Tn*1721* was the ∆Tn*21* element, which confers resistance to sulfonamides and streptomycin, residing within the substantial plasmid IncHI2 carried by strain X7020 (as illustrated in Figure 4b). Furthermore, four class 1 integron structures were identified, exhibiting the following gene cassette arrangements: (i) *dfrA12-aadA1-cmlA1-aadA2-qacI*; (ii) *dfrA17-aadA5*; (iii) *aac(6)-Ib-cr-bla*_OXA-1_-*catB3-orf1-orf2-sul1*; and (iv) *dfrA5*. The integron harboring the *dfrA17-aadA5* cluster demonstrated the classical 3′-conserved segment (3′-CS), characterized by a truncated *qac∆E1* and the *sul1* sulfonamide resistance gene. The larger assembly of *aac(6)-Ib-cr-bla*_OXA-1_-*catB3-orf1-orf2-sul1*, as previously mentioned, represented an atypical class 1 integron, whose 3′-CS segment was truncated due to the IS*Cr1* insertion.

**Figure 5:**
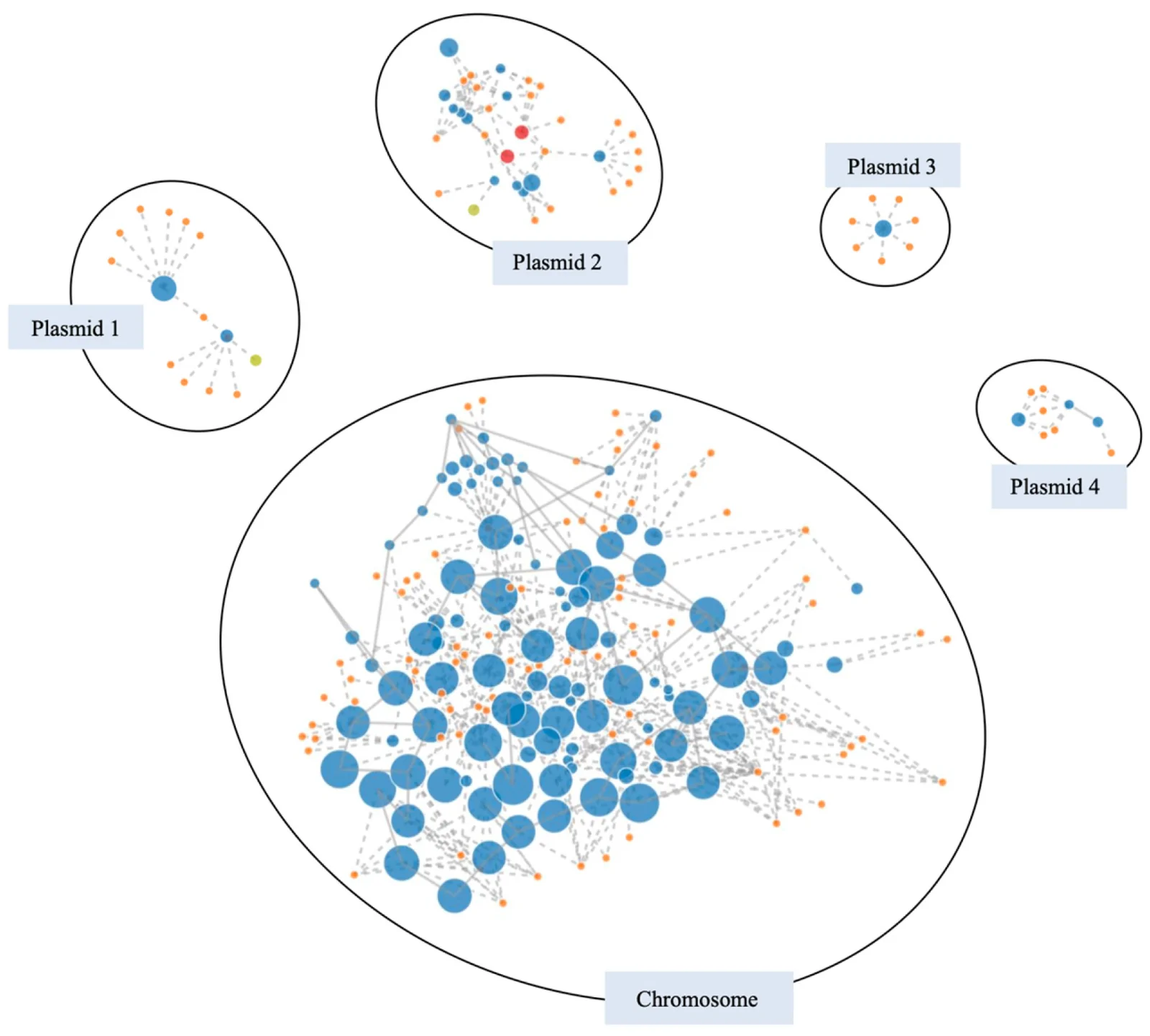
PLACNETw reconstruction of the *E. coli* X3071 genome. After manual pruning of the original network, four components were differentiated: the chromosome, plasmid 1 (IncX4 plasmid), plasmid 2 (IncFIA-FIB replicon), and plasmid 3 (IncR-IncFIA multi-replicon). Contigs are represented as blue nodes of size proportional to their length. Orange nodes indicate reference genomes. Contigs encoding plasmid relaxases and replication proteins are colored in red and yellow, respectively.

Furthermore, Figure 5 illustrates the PLACNETw reconstruction of the X3071 *E. coli* genome, wherein a complex network is distinctly observable in both chromosomes and plasmids. Plasmid 2 exhibited two MOB_F12_ relaxases (indicated by red nodes) and one IncX replicon type (represented by a yellow node). Additionally, an incomplete macrolide resistance cluster was identified, featuring a complete *mphA* gene and a truncated *mrx* gene, culminating in the genetic platform IS*26*-*mph*(A)-∆*mrx*-*orf477*-∆IS*Ecp1*-IS*26* located within plasmid 2. Two non-typeable and non-transposable plasmids were identified, with sizes ranging from 70 to 90 kb, and lacking the relaxase gene (specifically plasmids 3 and 4). Notably, the backbone of plasmid 3 demonstrated high homology (90% coverage, 100% identity) to plasmid pSvP1 (accession number NZ_MN510446), which has been characterized in a porcine ETEC isolate.

### 3.3. Phylogenomics

A phylogenetic tree using single-nucleotide polymorphisms (SNPs) in the core genome was created to explore potential host-associated adaptive mutations. To improve our understanding of evolutionary dynamics, additional E. coli genomes were selected from EnteroBase based on MLST criteria. Up to 32 *E. coli* genomes per sequence type, representing various sources and geographic regions, were obtained from the NCBI BioProjects database (Table S2). The final dataset for phylogenomic reconstruction, as illustrated in Figure S3, comprised 200 genomes, including those from our collection (n = 38). Among the total gene clusters (16,890), only 19.3% (3258) were shared across all genomes. This core genome illuminated the high genetic diversity of the studied collection, particularly underscored by the significant divergence of both sequence types and the differentiation observed within them, as categorized by the phylogenetic clustering identified by Clermont et al. [31].

**Figure 6:**
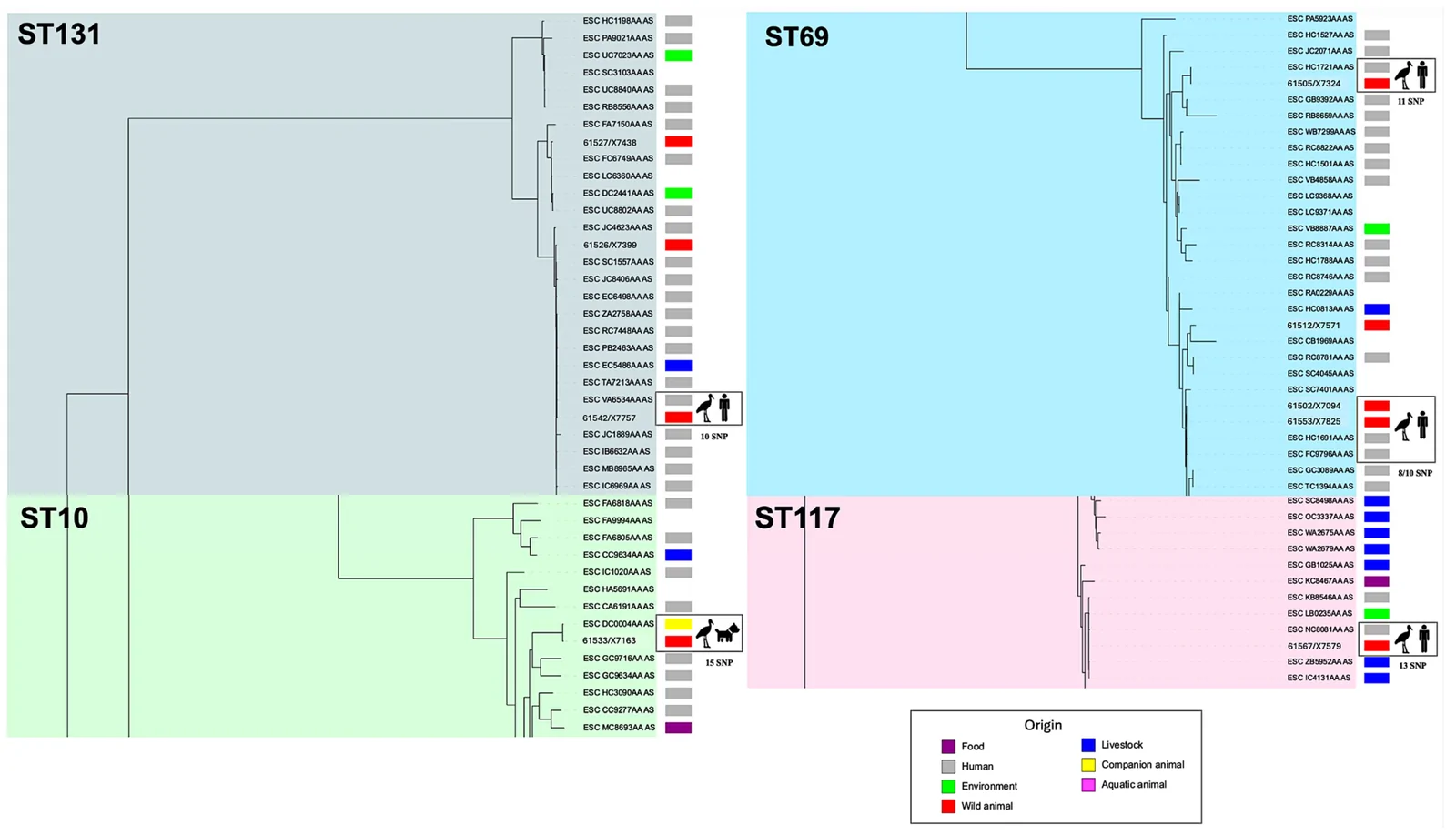
Summary of the four major branches of the core genome tree. Strongest relationships (SNP differences) that were observed between strains from multiple sources are highlighted in squares. Additionally, in the different branches, we observe the higher discriminatory power of WGS compared to conventional MLST typing.

As previously indicated, the core genome tree displayed remarkable consistency with the MLST data, as well as with the phylogroup distribution of our collection. Regarding the various clusters within the tree, a significant majority comprised strains isolated from diverse sources and geographically distant locations. Also, in numerous instances, the number of SNPs (Figure 6, Table S3) was lower between strains derived from different animal species compared to those within the same species. Several illustrative examples can be highlighted among some of the tree’s most populous branches (ST10, ST38, ST58, ST117, and ST131 groups). For instance, within the ST58 clade, characterized by an average of 312.8 SNPs among its constituents, the X7761 strain, isolated from a stork, exhibited the closest genetic relationship (58 SNPs) with the poultry-derived strain ESC_UC7253AA_AS (51 SNPs) (Table S3). A parallel observation was documented among members of the extensively distributed ST10 (average SNP: 678.8). The core genome of isolate X7163 (stork, Spain) exhibited a greater degree of similarity to ESC_DC0004AA (canine, USA) than to other *E. coli* strains derived from free-living hosts, revealing a mere 15 SNPs (Table S3). Notably, the exceptionally low count of SNPs (≤10) that distinguished our UPEC strains X7094 and X7825 (storks, Spain) from those associated with human hosts, specifically ESC_HC1691AA from Paraguay, which also belongs to the UPEC pathotype, is especially significant when considered against the average SNP count within sequence type 69 (ST69) (n = 925.8) (Figure 6). This observation was similarly reflected in the comparison of the genome of strain X7579 (stork, Spain) and that of ESC_NC8081AA (human, UK), where only 13 SNPs differentiated these two members within the primary ST117 cluster (average SNP = 359.6). A comparable pattern was observed for ST131 concerning strain X7757 and ESC_VA6534AA (human, Mexico) (Figure 6).

### 3.4. CRISPR/Cas Systems

The CRISPR/Cas and spacer repertoires were examined in the 38 *E. coli* strains. Systems I-E and I-F1 were detected in 32 genomes, predominantly I-E (n = 28, 87.5%), while I-E+I-F1 appeared in two genomes (5.3%).

Figure S4 illustrates the distinct genetic organizations associated with the I-E and I-F1 systems concerning the module situated between the two CRISPR arrays, which encompasses the *cas* gene set and the adjacent open reading frames (ORFs). A notable diversity of structures was observed within the I-E systems, as truncated elements IS*421*, IS*186*, or IS*30* were identified inserted within the *cas3* gene and adjacent to the *yqcE* gene in strains X7553, X2632, X3078, and X5726. Most strains were found to possess either the CRISPR/Cas I-E variant A or C, whereas strains X7760, X7823, X7581, X4879, X5102, and X3071 were associated with free-living animals (storks) and food-producing animals (pigs and food-derived products) and exhibited the absence of a complete copy of *cse2* (noted as variant A* in Figure S4).

Furthermore, the evolutionary relationship of the *cas* genes, inferred from the phylogenetic tree of the concatenated amino acid sequences of the *cas-E* genes, showed two clearly differentiated groups in the CRISPR/Cas I-E systems: (i) E1, consisting mainly of strains belonging to phylogroups A, C, B1, D, B2, and F; and (ii) E2, comprising mainly group A and D strains (Figure S5).

**Figure 7:**
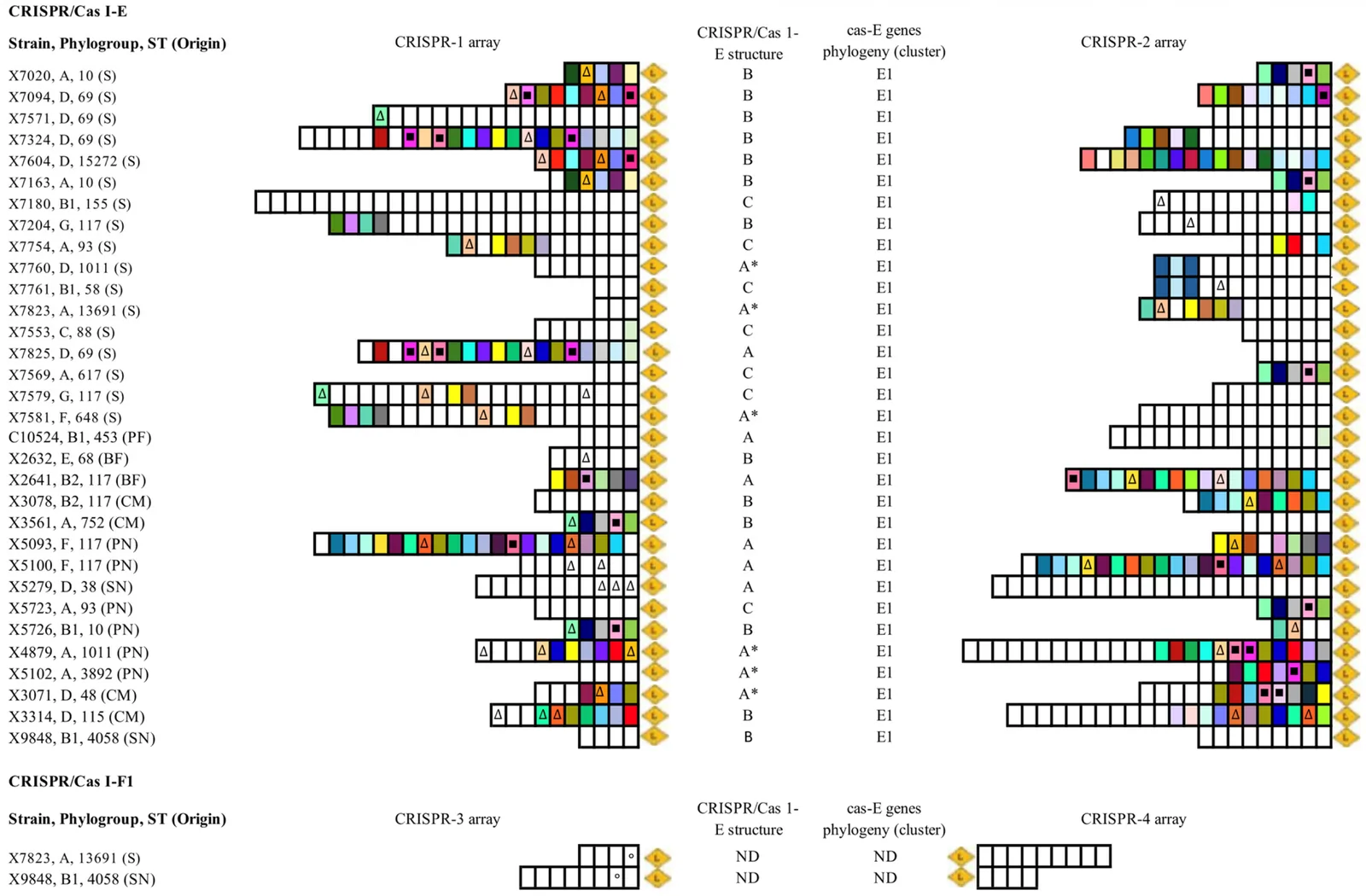
Outline of the CRISPR arrays located in the CRISPR/Cas I-E and I-F1 systems found in the genome of the *E. coli* collection. Spacers are represented as vertical boxes and are oriented towards the leader (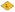). Boxes with the same color represent spacers appearing in ≥2 strains. There were no overlapping spacer sequences between CRISPR arrays 1, 2, 3 and 4, so each array’s colors should be interpreted independently. The different symbols indicate spacers that match at least 88% with phage (∆), plasmid (■) or IS-like sequences (∘) in databases. Origin: stork, fecal sample (S); pig farm, manure (PF); broiler farm (X2632, X2641 retrieved from manure); pig nostrils (PN); stork nestling, cloacal sample (SN); chicken meat (CM). CRISPR/Cas structures I-E (A, A*, B, C) and I-F1 (A), as well as cas gene clusters (E1, F), correspond to those depicted in Figures S1 and S2 of the Supplementary Materials, respectively. ND: not determined.

Concerning the CRISPR arrays, a total of 296, 361, and 12 spacers were identified in CRISPR-1, CRISPR-2, and CRISPR-3, respectively. Of these, 21%, 13% and 8% of spacers were noted to appear in more than one strain, irrespective of the clonal relationships of the bacteria (Figure 7 and Table 1). For the collection of *E. coli* examined, a substantial link was observed between the CRISPR-1/-2 and ST genetic lineages, albeit with some exceptions. We identified strains that shared the CRISPR-1 array, which were assigned to distinct sequence types (STs) and even to different phylogroups (X7825 and X4879). Furthermore, the CRISPR-3/-4 arrays exhibited a higher proportion of unique spacers compared to the CRISPR-1/-2 arrays (100% versus 35.2%).

Concerning the homology of spacer sequences to known genes (proto-spacers), we found significant differences between CRISPRs. Globally, among the 657 spacers present in CRISPR-1 to -3, 26 (4.0%) showed high homology (≥92%) to different mobile elements available in public databases. Precisely, in decreasing order of prevalence, 20 (3.1%), seven (1.1%), and one (0.2%) matched viral proto-spacers, plasmids (NZ_CP141089.1) and IS (IS*110* family), respectively.

**Limitations of the study:** The small sample size (n = 38) and the limited number of isolates in some pathotypes represent clear limitations. Additionally, the number of isolates between phenotypic ESBL-producing and non-ESBL strains weakens the statistical power needed to evaluate cross-talk between resistance mechanisms and specific virulence or fitness phenotypes. Since this isolate collection was built through a targeted screening based on fixed pathotypic markers rather than an unbiased random selection, these correlations characterize specific high-risk clonal platforms instead of reflecting broad epidemiological trends. Larger, systematic surveillance efforts across these One Health interfaces are still required to establish the generalizability of these observations.

## 4. Discussion

The genomic interplay between virulence factors, AMR, and CRISPR-Cas systems in *E. coli* is critical for understanding bacterial adaptability, pathogenicity, and the spread of resistance genes.

Regarding their virulence profile, we identified 47 virulence-associated genes, including key determinants for ExPEC and UPEC pathotypes. The in vitro adherence and invasion assays revealed that strains with ExPEC and UPEC genes showed the highest colonization rates, with significant differences among pathotypes depending on the phenotype analyzed. These findings align with previous studies and provide new insights into the link between genetic determinants and in vitro phenotypes. The elevated adherence and invasion capacity observed in ExPEC and UPEC strains align with previous reports demonstrating that these pathotypes possess a robust arsenal of virulence factors enabling the colonization of host tissues. Johnson et al. (2003) reported that UPEC strains commonly harbor fimbrial adhesins, such as P fimbriae (*pap*), which facilitate urinary tract colonization, while ExPEC strains possess a broader set of adhesins, including S and F1C fimbriae (*sfa*/*focDE*) and Dr-binding adhesins (*afa*/*draBC*) [32]. Our findings confirm these associations but further reveal that UPEC strains exhibit significantly higher invasion capacity than ExPEC. This suggests that, beyond fimbrial adhesins, other virulence determinants such as iron acquisition systems (*chuA*, *fyuA*) and toxins (*vat*) may play a critical role in the invasion of host cells, a phenomenon that has been less explored in previous studies.

Furthermore, the significant correlation between the ST117 lineage and increased invasion capacity observed in our study provides additional evidence for the pathogenic potential of this sequence type. In previous studies, we have detected that ST117 is emerging as a high-risk clone associated with extraintestinal infections in both humans and animals [8]. Our findings align with previous studies, demonstrating that ST117 *E. coli* strains are highly adept at invasiveness, likely due to the presence of virulence genes on their chromosomes. This arrangement may enhance the stability of these traits, making them easier to spread. Similarly, the high-risk lineage ST131 also exhibited notable invasive potential, corroborating prior research indicating its association with multidrug resistance and virulence [6]. In addition to adhesion and invasion, biofilm formation seems to represent an important virulence-associated trait in the strains analyzed. Biofilm-producing *E. coli* can persist in the urinary tract and on abiotic surfaces, increasing tolerance to antimicrobial treatment and host immune defenses, thereby contributing to chronic and recurrent infections. In our collection, biofilm formation was particularly relevant among ExPEC and UPEC isolates, reinforcing the multifactorial nature of their pathogenicity. High-risk clones such as ST131 have been consistently associated with enhanced biofilm production, which may further increase their pathogenic fitness and contribute to their global dissemination [33]. Beyond its contribution to persistence, the biofilm matrix may also play a mechanistic role in antimicrobial resistance by limiting antibiotic penetration and creating microenvironments that favor bacterial survival under subinhibitory concentrations. Moreover, biofilms can facilitate conjugative plasmid transfer, potentially enhancing the dissemination of ESBL and other AMR determinants identified in our strains. This interplay between biofilm formation, virulence, and horizontal gene transfer further supports the convergence of pathogenicity and multidrug resistance within these One Health reservoirs [34]. The comparative analysis of virulence gene associations aligns with established links between specific genes and pathotypes. As shown in Figure 4b, the *hra* gene was specifically associated with ExPEC strains, while *tsh* and *tia*, both implicated in adhesion and epithelial invasion, were uniquely linked to UPEC strains. These findings suggest that, beyond the core virulence gene repertoire, certain accessory factors contribute to pathotype-specific pathogenic mechanisms. ExPEC and UPEC strains showed improved adherence and invasion of epithelial cells, with UPEC strains having greater internalization. However, it is important to note that these associations reflect statistical correlations rather than direct phenotypic causation. The presence of a given gene pool does not inherently guarantee its transcriptional activation or translational execution under experimental conditions. Consequently, these genomic profiles are presented as markers that align with higher pathogenic trends, providing a baseline for future functional evaluation. Furthermore, our results support the strong association of *eae* with EPEC, *stx1/2* with STEC, and *eltAB-4* and *stap-STa1* with ETEC, consistent with previous genomic and functional studies [1].

Our analysis of 38 *E. coli* strains revealed diverse plasmid content, including size, replicon families, and plasmid numbers per isolate. Their presence in all strains highlights their role in bacterial adaptability, enabling the horizontal transfer of virulence and AMR determinants. Our findings align with previous reports demonstrating the widespread distribution of plasmids across diverse ecological niches, including wildlife [14,15], livestock [11,12,16], food-derived products [13], and clinical environments [35,36]. Small mobilizable plasmids were identified in nearly half (47.4%) of the strains, with most carrying only essential genes related to replication and mobility. The observed association between the relaxase subfamily MOB_Q12_ and a replication protein (*repL*) on pColE2 [col(156)] is consistent with prior studies highlighting the role of MOB_Q_ relaxases in plasmid dissemination among *Enterobacteriaceae* [37]. These plasmids are generally cryptic, lacking precise adaptive functions, but their persistence in bacterial populations suggests an evolutionary advantage yet to be fully elucidated. Previous studies have identified similar small plasmids in commensal and pathogenic *E. coli*, often carrying genes related to stress resistance and fitness, albeit at low frequencies [38]. As observed in our study, the cryptic nature of most of these plasmids reinforces the notion that their genetic content may contribute to bacterial survival under specific environmental pressures. Following this, the *iss* gene, frequently associated with avian colibacillosis and commonly found on pColV/BM plasmids, was detected in 84.2% of our strains. Sequence analysis revealed its clustering within the *iss* type 1 group, while a distinct sequence (termed type 13) was associated with lower serum survival, forming a subcluster within *iss* type 2. This differentiation in *iss* variants aligns with previous research indicating that specific *iss* alleles enhance bacterial resistance to host immune responses, particularly in avian pathogenic *E. coli* (APEC) [39]. The high prevalence of *iss* in our collection suggests a strong selective advantage conferred by this gene, particularly in strains colonizing wildlife and livestock reservoirs.

Larger plasmids were additionally detected as key hosts of ETEC-associated toxin genes (*stA*, *eltAB*) on broader IncF plasmids, consistent with prior findings [1]. The *stA1* toxin gene was found within the pURSta5726 plasmid (147,979 bp), embedded in a complex genetic structure containing virulence determinants (*hlyABCD*, *tpx*) and metal resistance genes. Similarly, the pUREtlAB5726 plasmid (101,609 bp) carried *eltA* and *eltB*, alongside a series of insertion sequences and regulatory elements such as *marA* and *hlyC*. The presence of toxin genes on IncF plasmids aligns with previous reports indicating that these plasmids serve as key vectors for virulence factor dissemination among pathogenic *E. coli* lineages [40]. Furthermore, our study reinforces the notion that ETEC strains rely on plasmid-borne toxin genes for their pathogenicity, emphasizing the role of mobile genetic elements in the evolution of diarrheagenic *E. coli*. In previous studies, we revealed the role of plasmids in disseminating critically important AMR genes such as *bla*_ESBL_, *bla*_CP,_ or *bla*_pAmpC_ [11,12,13,14,15,16]. Following previous studies [14,15,41,42], we report the predominance of conjugative IncF (MOB_F12_) and IncI1 (MOB_P12_) plasmids, consistent with earlier reports, as the leading carriers of AMR genes in *E. coli*. The presence of multiple Inc groups within a single strain suggests that *E. coli* in wildlife and livestock reservoirs may serve as essential sources of multidrug-resistant (MDR) bacteria. Our investigation into other critical AMR determinants highlighted the diverse genetic contexts of *bla*_TEM-1_ variants, which were identified within different transposon-associated environments. The most prevalent *bla*_TEM-1B_ variant was found upstream of the *tnpR* gene of Tn*1331*, while the *bla*_TEM-234_ variant was embedded within a composite transposon containing IS*15*, IS*1*, and IS*150*. This variation in genetic environments supports the hypothesis that mobile genetic elements drive the continuous reshuffling and dissemination of resistance determinants in *E. coli* [43]. Additionally, the identification of complex integron structures, such as the *aac(6)-Ib-cr-bla*_OXA-1_*-catB3-orf1-orf2-sul1* cassette, underscores the role of integrons in conferring multidrug resistance through horizontal gene transfer.

The phylogenomic analysis conducted in this study provides valuable insights into the evolutionary relationships and host-associated adaptation of *E. coli* strains from diverse origins. The phylogenetic clustering observed in our core genome tree exhibited strong concordance with our strains’ MLST-based classification and phylogroup distribution, aligned with previous studies that have demonstrated the robustness of MLST in capturing *E. coli* population structure [44]. Our study found lower SNP divergence between strains from different animal species compared to those from the same species. This suggests that certain *E. coli* lineages may show host generalism, retaining genetic similarity despite geographic and ecological variations, as seen with ST10, ST58, ST117, and ST131 [45]. One of the most striking observations in our study was the exceptionally low SNP divergence (≤10 SNPs) between certain wildlife-associated *E. coli* strains and human-derived strains (associated in most cases with UPEC pathotypes). For instance, our UPEC strain, X7579 (stork, Spain), exhibited only 13 SNP differences from ESC_NC8081AA (a UPEC *E. coli* human strain from the UK) within the ST117 cluster, providing compelling evidence of the zoonotic potential of specific *E. coli* lineages circulating in human and animal reservoirs [15,46,47].

These findings confirm that *E. coli* is a dynamic species that crosses host barriers, highlighting the importance of environmental and wildlife reservoirs in the persistence and spread of clinically relevant *E coli*. Given that high-risk STs such as ST117 and ST131 were among those exhibiting interspecies transmission, it is plausible that these lineages may serve as key vectors in the spread of virulence and antimicrobial resistance determinants between human and animal populations. For this reason, WGS approaches combined with transcriptomic and functional assays could help elucidate whether specific SNPs contribute to host adaptation, resistance acquisition, or niche specialization.

The architectural presence of Clustered Regularly Interspaced Short Palindromic Repeats (CRISPRs) and their associated *cas* genes has been documented in bacteria and archaea since the late 20th century [9,10]. In *Escherichia coli*, these systems predominantly belong to subtypes I-E and I-F1. Subtype I-E typically encompasses two CRISPR arrays (CRISPR-1 and CRISPR-2) and eight *cas* genes, whereas subtype I-F1 is characterized by six *cas* genes flanked by CRISPR-3 and CRISPR-4 arrays [48,49]. Genomic profiling in this study revealed that 87.5% of the analyzed *E. coli* isolates harbored the CRISPR/Cas I-E system, while 5.3% carried the I-F1 subtype. This high prevalence aligns with the established ubiquity of these defense platforms across the species [50].

However, a critical genomic feature observed in this collection is the structural disruption of these systems, marked by the insertion of IS elements within the *cas3* gene, likely favored by the presence of GC-rich target regions within these loci. Structurally broken *cas* clusters could limit the cell’s interference capacity, potentially facilitating the acquisition of resistance plasmids and driving the evolution of MDR clones [9,51]. Nevertheless, it is crucial to emphasize that these structural insights are derived strictly from in silico predictions. A major caveat of this approach is that genomic architecture alone cannot definitively determine functional outcomes; transcriptomic or phenotypic assays are necessary to verify whether these specific IS insertions completely abrogate immunity or if low-level, uncharacterized restriction mechanisms still persist. The spacer analysis demonstrated substantial diversity, with the CRISPR-3/-4 arrays exhibiting complete uniqueness (100%) compared to CRISPR-1/-2 (35.2%). Given the established role of unique spacers in adaptive immunity [52], these differences indicate that distinct environmental and ecological pressures shape specific arrays across One Health interfaces. Furthermore, only 4% of the total spacer repertoire matched known mobile genetic elements in public databases, a finding that supports the hypothesis that modern MDR strains may circumvent or lack active CRISPR interference against currently circulating mobilomes [9]. However, this low homology must be interpreted with caution and treated as a hypothesis-generating observation, as database limitations and the descriptive nature of bioinformatic mapping preclude definitive conclusions regarding real-time plasmid or phage restriction. In terms of evolutionary history, the phylogenetic tree inferred from the concatenated amino acid sequences of the *cas*-E and *cas*-F1 genes resolved the I-E systems into two major clades (E1 and E2), with minor variations in the I-F1 cluster, consistent with previously reported evolutionary lineages [53]. Finally, by integrating CRISPR-Cas structural variability, virulence factor profiles, plasmid-borne resistance, and phylogenomic data, this study provides a more comprehensive view of *E. coli* genomic dynamics. Uncovering these underlying genetic linkages across animal and food reservoirs underscores the practical necessity of genomic monitoring within One Health frameworks to track and mitigate the dissemination of high-risk MDR clones. Future research expanding into wet-lab functional assays will be essential to translate these bioinformatic observations into targeted infection control and antimicrobial stewardship strategies.

## 5. Conclusions

The integration of genomic and in vitro data in this study establishes potential links between specific core/accessory virulence markers and phenotypic colonization traits across diverse One Health reservoirs. Phenotypic assays validated that the accessory gene pool determines pathotype-specific behaviors, with *hra* and *tsh/tia* emerging as key determinants driving the epithelial adherence and internalization observed in ExPEC and UPEC isolates, respectively. These virulence networks, combined with the biofilm-forming capacity, are rooted in emerging high-risk clonal lineages such as ST117 and ST131, potentially enhancing their persistence and success outside conventional clinical niches. The close phylogenetic proximity (≤10–13 SNPs) detected between wild-bird (stork) isolates and human reference genomes confirms interspecies transmission and clonal circulation across environmental interfaces, demonstrating that wildlife acts as a dynamic reservoir for virulent *E. coli*. Structurally, while conjugative IncF and IncI1 plasmids remain the primary vectors sustaining MDR resistance phenotypes, genomic screening reveals that these MDR isolates frequently exhibit disrupted or compromised CRISPR-Cas subtype I-E systems due to insertion sequences in the cas3 locus. Finally, these findings highlight that tracking virulence plasticity alongside clonal distribution via WGS may be recommended within One Health networks to monitor and anticipate the expansion of high-risk zoonotic pathogens.

## Figures and Tables

**Table 1:** Genetic features of *Escherichia coli* strains from different sources based on in silico Whole-Genome Sequence analysis.

Strain (Origin) ^a^	Plasmid Typing			Virulence Genes			CRISPR/Cas Systems			Antimicrobial Resistance Genes		ST/PG
	No. Plasm.	Inc/Rep Types, Phages	Relaxes Protein	Pathotype ^b–f^	Chromosome	Plasmids	Subtype	No. Spacers	Class	Chromosome	Plasmids	
**X2583** **(BF-a)**	3	Col8282, ColpVC, IncFIB	MOB_F12_	EXPEC	*aslA*, *air*, ***chuA***** ^a^**, *eilA*, *fimH*, *gad*, *hlyE*, *hra*, *iha*, *iss*, *kpsE*, ***kpsMII***** ^b^**, *nlpI*, ***papC***** ^b^**, *shiA*, *shiB*	*anr*, *cba*, *cea*, *cia*, *cma*, *colE2-like*, *colE9*, *csgA*, *etsC*, *fdeC*, *hlyF*, *iucC*, ***iutA*** **^b^**, *ompT*	-	-	-	-	*bla*_SHV-12_, *aadA1*, *aadA2*, *cmlA1*, *sul3*, *tet*(A)	770/E
**X2632** **(BF-m)**	1	IncHI2	MOB_H11_	EXPEC/UPEC	*aslA*, *air*, *astA*, ***chuA***** ^a^**, *eilA*, *espY2*, *fimH*, ***fyuA***** ^a^**, *gad*, *hlyE*, *irp2*, *kpsE*, ***kpsMII***** ^b^**, *lpfA*, *mchB*, *mchC*, *nlpI*, *terC*, *yehA*, *yehB*, *yehC*, *yehD*	*csgA*, *fdeC*, *mchF*, ***iutA***** ^b^**, *sitA*, *traT*	I-E	6, 6	B	-	*bla*_SHV-12_, *aadA1*, *aadA2*, *cmlA1*, *sul3*	68/E
**X2641** **(BF-m)**	1	IncHI2	MOB_H11_	UPEC	*aslA*, ***chuA***** ^a^**, *cvaC*, *fimH*, ***fyuA***** ^a^**, *gad*, *iha*, *ireA*, *iss*, *lpfA*, *nlpI*, *pic*, *shiA*, *terC*, *tia*, *tsh*, ***vat***** ^a^**, *yehA*, *yehB*, *yehC*, *yehD*	*anr*, *csgA*, *etsC*, *fdeC*, *hlyF*, *iroN*, *irp2*, *iucC*, ***iutA***** ^b^**, *ompT*, *sitA*, *traJ*, *traT*	I-E	6, 17	A	-	*bla*_SHV-12_, *aadA1*, *aadA2*, *cmlA1*, *sitABCD*, *sul3*	117/B2
**X3078** **(CM)**	4	ColpVC, IncFIB, IncI1, IncX1	MOB_F12_, MOB_P12_, MOB_P3_	UPEC	*aslA*, *air*, ***chuA***** ^a^**, *eilA*, *fimH*, *gad*, *hlyE*, *hra*, *iha*, *kpsE*, ***kpsMII***** ^b^**, *nlpI*, ***papC***** ^b^**, *shiA*, *shiB*	*cba*, *cea*, *cia*, *cma*, *csgA*, *etsC*, *fdeC*, *hlyF*, *iroN*, *iss*, *iucC*, ***iutA***** ^b^**, *ompT*, *sitA*, *traJ*, *traT*	I-E	7, 10	B	-	*bla*_CTX-M-1_, *bla*_TEM-1B_, *aac(3)-IIa*, *aadA1*, *aph(3″)-Ib*, *aph(6)-Id*, *dfrA1*, *sul2*, *tet*(A)	117/B2
**X3561** **(CM)**	2	ColpVC, p0111	-	EPEC	*aslA*, *astA*, ***eae-b01a-beta***** ^c^**, *espA*, *espB*, *espF*, *fimH*, *gad*, *hha*, *hlyE*, *nleB*, *nlpI*, *terC*, *tia*, *tir*, *yehA*, *yehB*, *yehC*, *yehD*	*anr*, *cia*, *cif*, *csgA*, *fdeC*, *mchF*, *traJ*, *traT*	I-E	5, 6	B	-	*bla*_TEM-52_, *aph(3″)-Ib*, *aph(6)-Id*	752/A
**C10524** **(PF-m)**	3	Col156, IncFIA-IncFIB, p0111	MOB_F12_	EXPEC	*astA*, ***fyuA***** ^a^**, *gad*, *hlyE*, *irp2*, *iss*, *kpsE*, ***kpsMII***** ^b^**, *lpfA*, *papC*, *senB*, *terC*, *traT*	*cea*, *iucc*, ***iutA***** ^b^**, *sitA*, *traT*	I-E	4, 15	A	-	*bla*_CTX-M-14_, *bla*_TEM-1B_, *aac(3)-IId*, *aadA5*, *aph(3″)-Ib*, *aph(6)-Id*, *dfrA17*, *erm*(B), *mph*(A), *qacE*, *sul1*, *sul2*, *tet*(A)	453/B1
**X5093** **(PN)**	4	IncFIB, IncI1, IncN, IncR	MOB_F12_, MOB_P12_, MOB_F11_	EXPEC/UPEC	*AslA*, *astA*, ***chuA***** ^a^**, ***fyuA***** ^a^**, *hha*, *irp2*, *iss*, *lpfA*, *nlpI*, ***papA***** ^b^, *papC* ^b^**, *pic*, *shiB*, *terC*, *tsh*, *vat*, *yehB*, *yehC*, *yehD*	*anr*, *cea*, *cma*, *csgA*, *fdeC*, *hlyF*, *iroN*, *irp2*, *iucC*, ***iutA***** ^b^**, *mcbA*, *mchF*, *sitA*, *traJ*	I-E	24, 8	A	-	*bla*_CTX-M-14_, *bla*_TEM-1B_, *aadA1*, *catA1*, *dfrA1*, *qnrS1*, *sitABCD*, *sul3*, *tet*(A)	117/F
**X5726** **(PN)**	5	Col(E10), IncFIB-IncFIC, IncHI2, IncI1, IncY	MOB_F12_, MOB_H11_, MOB_P12_	ETEC	*aslA*, *fedA*, *fedB*, *fedE*, *fedF*, *fimH*, ***fyuA***** ^a^**, *hha*, *hlyA*, *hlyE*, *iha*, *irp2*, *neuC*, *nlpI*, *terC*, *yehA*, *yehB*	*anr*, *cea*, *colE2*, *csgA*, ***eltIAB-4***** ^d^**, ***estap-STa1***** ^d^**, *fimA*, *traT*	I-E	5, 4	B	-	*bla*_CTX-M-65_, *bla*_OXA-10_, *bla*_TEM-70_, *aac(3)-IV*, *aadA2*, *aadA24*, *aph(3″)-Ib*, *aph(3′)-Ia*, *aph(4)-Ia*, *aph(6)-Id*, *ARR-3*, *cmlA1*, *dfrA1*, *dfrA14*, *floR*, *lnu(F)*, *mcr-4.5*, *qnrS1*, *sul3*, *tet*(A), *tet*(B)	10/B1
**X5279** **(SN)**	4	Col(pVC), IncFIB-IncFII, IncX1, IncY	MOB_F12_, MOB_P3_	EXPEC	*aslA*, *air*, *astA*, ***chuA***** ^a^**, *cvaC*, *eilA*, *fimH*, *gad*, *hlyE*, *hra*, *iha*, *iss*, *kpsE*, ***kpsMII***** ^b^**, *nlpI*, *shiA*, *terC*, *tia*, *yehA*, *yehB*, *yehC*, *yehD*	*anr*, *cba*, *cea*, *cia*, *cma*, *csga*, *etsC*, *fdeC*, *hlyF*, *iucC*, ***iutA***** ^b^**, *ompT*, *sitA*, *traJ*, *traT*	I-E	11, 23	A	-	*bla*_SHV-12_, *aadA1*, *aadA2b*, *aph(6)-Id*, *cmlA1*, *lnu(F)*, *qnrS1*, *sul3*	38/D
**X7017** **(S)**	1	IncI1	MOB_P12_	EPEC	*aslA*, *chuA*, ***eae-e06-η* ^c^**, *espA*, *espC*, *espF*, *espJ*, *fimH*, *gad*, *hha*, *nleB*, *nleC*, *nlpI*, *shiB*, *terC*, *tir*, *yehA*, *yehB*, *yehC*, *yehD*, ***yfcV*** **^a^**	*-*	-	-	-	-	*bla*_SHV-12_	788/B2
**X7020** **(S)**	1	IncHI2	MOB_H11_	EPEC	*aslA*, ***eae-e01-ε***** ^c^**, *espA*, *espB*, *espF*, *fimH*, *hha*, *hlyE*, *nleA*, *nleB*, *nleC*, *nlpI*, *sepA*, *tccP*, *terC*, *tir*, *yehA*, *yehB*, *yehC*, *yehD*	*-*	I-E	4, 4	B	-	*bla*_CTX-M-65_, *bla*_TEM-70_, *aac(3)-IV*, *aadA2*, *aadA24*, *aph(3″)-Ib*, *aph(3′)-Ia*, *aph(4)-Ia*, *aph(6)-Id*, *cmlA1*, *floR*, *lnu*(F), *qnrS1*, *sul2*, *sul3*, *tet*(A)	10/A
**X7094** **(S)**	1	IncY	-	UPEC	*aslA*, *air*, ***chuA***** ^a^**, *eilA*, *fimH*, ***fyuA***** ^a^**, *gad*, *hha*, *hlyE*, *irp2*, *kpsE*, ***kpsMIII***** ^b^**, *lpfA*, *nlpI*, *terC*, *yehA*, *yehB*, *yehC*, *yehD*	*anr*, *ompT*, *sitA*, *traJ*, *traT*	I-E	8, 8	B	-	*bla*_CTX-M-15_, *bla*_TEM-1B_, *aac(3)-IId*, *aph(3″)-Ib*, *aph(6)-Id*, *dfrA14*, *qnrS1*, *sul2*, *sul3*, *tet*(A)	69/D
**X7104** **(S)**	2	IncFIB, IncI1	MOB_F12_, MOB_P12_	EXPEC/UPEC	*aslA*, *air*, ***chuA*** **^a^**, *cib*, *cvaC*, *eilA*, *espY2*, *fimH*, *gad*, *hha*, *hlyE*, *hra*, *iss*, *kpsE*, ***kpsMII*** **^b^**, *lpfA*, *nlpI*, ***papA*** **^b^**, ***papC*** **^b^**, *shiB*, *terC*, *yehA*, *yehB*, *yehC*, *yehD*, ***yfcV*** **^a^**	*anr*, *cea*, *cib*, *cma*, *csgA*, *fdeC*, *iucC*, ***iutA*** **^b^**, *ompT*, *sitA*, *traJ*, *traT*	-	-	-	*bla*_CTX-M-55_	*bla*_TEM-1B_, *aph(3″)-Ib*, *aph(3′)-Ia*, *aph(6)-Id*, *sitABCD*, *sul2*, *tet*(A)	457/F
**X7324** **(S)**	3	Col(156) (2), IncFIA, IncI1, IncY	MOB_F12_, MOB_P12_	EXPEC/UPEC	*aslA*, *air*, ***chuA***** ^a^**, *eilA*, *fimH*, ***fyuA***** ^a^**, *gad*, *hha*, *hlyE*, *irp2*, *iss*, *kpsE*, *lpfA*, *nlpI*, ***papA***** ^b^**, ***papC***** ^b^**, *senB*, *terC*, *yehA*, *yehB*, *yehC*, *yehD*	*anr*, *csgA*, *fdeC*, *hlyF*, *ompT*, *sitA*, *traT*	I-E	8, 15	B	-	*bla*_DHA-1_, *dfrA17*, *mph*(A), *qacE*, *qnrB4*, *sul2*	69/D
**X7571** **(S)**	3	IncB/O/K/Z, IncFIA-FIB-FIC(FII), IncX1	MOB_P12_, MOB_F12_, MOB_P3_	EXPEC/UPEC	*aslA*, *air*, ***chuA***** ^a^**, *cvaC*, *eilA*, *fimH*, ***fyuA***** ^a^**, *gad*, *hha*, *hlyE*, *iha*, *irp2*, *iss*, *kpsE*, ***kpsMII***** ^b^**, *lpfA*, *mchB*, *mchC*, *neuC*, *nlpI*, *shiA*, *shiB*, *terC*, *tsh*, *yehA*, *yehB*, *yehC*, *yehD*	*anr*, *csgA*, *cvaC*, *etsC*, *hlyF*, *iroN*, *iucC*, ***iutA***** ^b^**, *mchF*, *ompT*, *sitA*, *traT*, *tsh*	I-E	16, 7	B	-	*bla*_CTX-M-14_, *aadA5*, *aph(3″)-Ib*, *aph(6)-Id*, *dfrA17*, *qacE*, *sitABCD*, *sul2*, *tet*(A)	69/D
**X7604** **(S)**	3	Col156, ColpVC, IncFIB-IncFII	MOB_F12_	EXPEC/UPEC	*aslA*, *air*, ***chuA***** ^a^**, *eilA*, *fimH*, ***fyuA***** ^b^**, *gad*, *hha*, *hlyE*, *iha*, *irp2*, *iss*, *kpsE*, ***kpsMII***** ^b^**, *lpfA*, *nlpI*, ***papA***** ^b^**, *sat*, *senB*, *terC*, *yehA*, *yehB*, *yehC*, *yehD*	*csgA*, *fdeC*, *irp2*, *iucC*, ***iutA***** ^b^**, *ompT*, *sitA*, *traT*	I-E	6, 17	B	*bla*_CTX-M-15_	*bla*_CTX-M-15_, *bla*_TEM-1B_	15,272/D
**X7399** **(S)**	2	IncFIA-FII, IncI1	MOB_F12_, MOB_P12_	EXPEC/UPEC	*aslA*, ***afaA***** ^b^, *afaC* ^b^, *afaD* ^b^**, ***chuA***** ^a^**, *fimH*, ***fyuA***** ^a^**, *hha*, *iha*, *kpsE*, ***kpsMII***** ^b^**, *nfaE*, *nlpI*, ***papA***** ^b^**, *sat*, *shiA*, *terC*, *usp*, *yehB*, *yehC*, *yehD*, ***yfcV***** ^a^**	*cia*, *csgA*, *fdeC*, *irp2*, *iss*, *iucC*, ***iutA***** ^b^**, *ompT*, *sitA*, *traJ*, *traT*	-	-	-	-	*bla*_CTX-M-15_, *bla*_TEM-1B_, *aadA5*, *dfrA17*, *mph*(A), *qacE*, *sul2*	131/B2
**X7438** **(S)**	2	IncFIB, IncI1	MOB_F12_, MOB_P12_	EXPEC/UPEC	*aslA*, ***chuA***** ^a^**, *cnf1*, *dhaK*, *fimH*, ***fyuA***** ^a^**, *gad*, *hha*, *hlyA*, *hra*, *ibeA*, *iha*, *irp2*, *iss*, *kpsE*, ***kpsMII_K5***** ^b^**, *mchB*, *mchC*, *nlpI*, ***papA***** ^b^**, ***papC***** ^b^**, *shiA*, *shiB*, *terC*, *usp*, *yehA*, *yehB*, *yehC*, *yehD*, ***yfcV***** ^b^**	*anr*, *etsC*, *etsC*, *hlyF*, *iucC*, ***iutA***** ^b^**, *ompT*, *sitA*, *traJ*, *traT*	-	-	-	-	*bla*_CTX-M-32_, *bla*_TEM-1A_, *aadA1*, *aadA2*, *cmlA1*, *dfrA12*, *dfrA17*, *sitABCD*, *sul3*, *tet*(A)	131/B2
**X7163** **(S)**	3	Col156, IncFIA-IncFII, IncR	MOB_F12_	EXPEC	*aslA*, *astA*, ***fyuA***** ^a^**, *hlyE*, *irp2*, *nlpI*, *senB*, *terC*, *yehA*, *yehB*, *yehC*, *yehD*	-	I-E	5, 3	B	-	*bla*_CTX-M-55_, *bla*_TEM-216_, *aac(3)-IId*, *erm*(B)	10/A
**X7180** **(S)**	3	IncB/O/K/Z, IncFIB-FIC(FII), IncI1	MOB_P12_, MOB_F12_, MOB_P12_	UPEC	*fimH*, *gad*, *hha*, *hlyE*, *iss*, *lpfA*, *nlpI*, *shiA*, *terC*, *tia*, *yehA*, *yehB*, *yehC*, *yehD*	*anr*, *cib*, *cma*, *hlyF*, *iroN*, *iucC*, ***iutA***** ^b^**, *ompT*, *sitA*, *traJ*, *traT*, *tsh*	I-E	26, 12	C	-	*bla*_CMY-2_, *bla*_CTX-M-1_, *sitABCD*, *sul2*, *tet*(A)	155/B1
**X7204** **(S)**	3	IncFIB, IncI1, IncX3	MOB_F12_, MOB_P12_, MOB_P3_	UPEC	*aslA*, ***chuA***** ^a^**, *cib*, *cvaC*, *fimH*, *iha*, *ireA*, *lpfA*, *nlpI*, *pic*, *terC*, ***vat***** ^a^**, *yehA*, *yehB*	*iss*	I-E	21, 13	B	-	*bla*_SHV-12_, *bla*_TEM-1B_, *aadA1*, *dfrA1*, *qnrS1*, *sitABCD*, *sul2*	117/G
**X7754** **(S)**	2	IncFIB, p0111	MOB_F12_	EXPEC/UPEC	*aslA*, *fimH*, *gad*, *gad*, *hlyE*, *iha*, *kpsE*, ***kpsMII***** ^b^**, *mchB*, *mchC*, *neuC*, *nlpI*, *shiA*, *terC*, *terC*, *yehA*, *yehB*, *yehC*, *yehD*	*anr*, *cma*, *etsC*, *etsC*, *hlyF*, *iucC*, ***iutA***** ^b^**, *mchF*, *sitA*	I-E	13, 6	C	-	*bla*_SHV-12_, *sitABCD*, *tet*(A)	93/A
**X7757** **(S)**	2	Col156, IncFIA-IncFIB	MOBF12	EXPEC	*aslA*, ***chuA***** ^a^**, *cnf1*, *fimH*, ***fyuA***** ^a^**, *gad*, *hha*, *hlyA*, *hra*, *iha*, *irp2*, *kpsE*, ***kpsMII***** ^b^**, *nlpI*, ***papA***** ^b^**, ***papC***** ^b^**, *sat*, *senB*, *shiB*, *terC*, *usp*, *yehA*, *yehB*, *yehC*, *yehD*, ***yfcV***** ^a^**	*anr*, *csgA*, *fdeC*, *irp2*, *iss*, *iucC*, *ompT*, *traJ*, *traT*	-	-	-	*bla*_CTX-M-15_	*bla*_OXA-1_, *aac(3)-IIa*, *aac(6′)-Ib-cr*, *aac(6′)-Ib-cr*, *aadA5*, *catB3*, *dfrA17*, *mph*(A), *qacE*, *sul1*, *tet*(A)	131/B2
**X7760** **(S)**	1	IncI1	MOB_P12_	-	*aslA*, *air*, *astA*, ***chuA***** ^a^**, *eilA*, *espY2*, *fimH*, *gad*, *hlyE*, *hra*, *iss*, *nlpI*, *ompT*, *terC*, *yehB*, *yehC*, *yehD*	*csgA*, *fdeC*, *ompT*	I-E	7, 12	A *	-	*bla*_SHV-12_, *bla*_TEM-1B_, *aac(3)-IId*, *aadA1*, *aadA2*, *catA1*, *cmlA1*, *dfrA12*, *mph*(A), *qacE*, *sul1*, *sul3*	1011/D
**X7761** **(S)**	5	IncFIB-IncFII, IncI1, IncQ1, IncX4, IncY	MOB_F12_, MOB_P12_	EXPEC	*cvaC*, *fimH*, ***fyuA***** ^a^**, *gad*, *hlyE*, *iroN*, *iss*, ***iutA***** ^b^**, ***kpsMII***** ^b^**, *lpfA*, *nlpI*, *sitA*, *terC*, *traJ*, *traT*, *yehA*, *yehB*, *yehC*, *yehD*	*anr*, *cea*, *cia*, *csgA*, *etsC*, *fdeC*, *hlyF*, *irp2*, *iucC*, ***iutA***** ^b^**, *mchF*, *ompT*	I-E	8, 15	C	-	*bla*_CTX-M-14_, *bla*_CTX-M-55_, *bla*_TEM-1B_	58/B1
**X7823** **(S)**	2	IncFIB(K), IncX1	MOB_F12_, MOB_P3_	-	*fimH*, *hha*, *hlyE*, *mrkA*, *nlpI*, *terC*, *yehA*, *yehB*, *yehD*	*csgA*, *fdeC*, *iss*	I-E, I-F1	3, 13, 8	A *	-	*bla*_CTX-M-15_, *bla*_TEM-1B_, *aph(3″)-Ib*, *aph(6)-Id*, *dfrA14*, *qnrS1*, *sul2*, *tet*(A)	13,691/A
**X7825** **(S)**	2	IncHI1, IncY	MOB_H12_	UPEC	*aslA*, *air*, ***chuA* ^a^,***clpK2*, *eilA*, *fimH*, ***fyuA***** ^a^**, *gad*, *hha*, *hlyE*, *irp2*, *iss*, *kpsE*, ***kpsMIII***** ^b^**, *lpfA*, *nlpI*, *terC*, *yehA*, *yehB*, *yehC*, *yehD*	*csgA*, *estC*, *fdeC*, *irp2*, *ompT*, *sitA*	I-E	8, 8	A	-	*bla*_CTX-M-15_, *bla*_TEM-1B_, *aph(3″)-Ib*, *aph(6)-Id*, *qnrS1*, *sul2*, *tet*(A)	69/D
**X7553** **(S)**	3	Col(MG828), IncFIB-FIC(FII)-FII, IncI1	MOB_F12_, MOB_P12_	EXPEC	*cvaC*, *fimH*, ***fyuA***** ^a^**, *gad*, *hha*, *hlyE*, *irp2*, *iss*, *lpfA*, *nlpI*, ***papC***** ^b^**, *terC*, *yehA*, *yehB*, *yehC*, *yehD*	*anr*, *cba*, *cib*, *cma*, *cvaC*, *etsC*, *hlyF*, *iroN*, *iucC*, ***iutA***** ^b^**, *mchF*, *ompT*, *sitA*, *traJ*, *traT*, *tsh*	I-E	7	C	-	*bla*_CTX-M-1_, *aadA5*, *catA1*, *dfrA17*, *sul2*, *tet*(A)	88/C
**X7569** **(S)**	1	IncFIA-IncFIB	MOB_F12_	-	*aslA*, ***fyuA***** ^a^**, *gad*, *hha*, *hlyE*, *iss*, *terC*, *tsh*, *yehA*, *yehB*, *yehC*, *yehD*	*anr*, *cea*, *colE2*, *csgA*, *etsC*, *fdeC*, *hlyF*, *iroN*, *irp2*, *iucC*, ***iutA***** ^b^**, *mchF*, *ompT*, *sitA*, *traT*	I-E	3, 5	C	*bla*_CTX-M-15_	*bla*_TEM-1B_, *aph(3″)-Ib*, *aph(6)-Id*, *dfrA14*, *sitABCD*, *sul2*, *tet*(A)	617/A
**X7579** **(S)**	2	IncFIA-IncFIB, IncX1	MOB_F12_, MOB_P3_	UPEC	*aslA*, ***chuA***** ^a^**, *cvaC*, *fimH*, ***fyuA***** ^a^**, *gad*, *hha*, *iha*, *ireA*, *iss*, *lpfA*, *mchB*, *mchC*, *neuC*, *nlpI*, *pic*, *terC*, *vat*, *yehB*, *yehC*, *yehD*	*anr*, *cea*, *cia*, *cma*, *csgA*, *etsC*, *fdeC*, *hlyF*, *iroN*, ***iutA***** ^b^**, *mchF*, *ompT*, *sitA*, *traJ*, *traT*	I-E	22, 8	C	-	*bla*_CTX-M-1_, *sitABCD*	117/G
**X7581** **(S)**	2	IncFIA-IncFIB, IncY	MOB_F12_	UPEC	*aslA*, *air*, ***chuA***** ^a^**, *eilA*, *espY2*, *fimH*, ***fyuA***** ^a^**, *gad*, *hlyE*, *iss*, *kpsE*, *lpfA*, *nlpI*, *terC*, *yehB*, *yehC*, *yehD*, ***yfcV***** ^a^**	*anr*, *csgA*, *fdeC*, *irp2*, ***KpsMII***** ^b^**, *ompT*, *traT*	I-E	21, 13	A *	*bla*_CTX-M-15_, *Inu(F)*	*bla*_OXA-1_, *bla*_TEM-1B_, *aac(6′)-Ib-cr*, *aac(6′)-Ib-cr*, *catB3*, *lnu(F)*, *tet*(A)	648/F
**X5100** **(PN)**	3	IncI1, IncN, IncR	MOB_P12_, MOB_F11_	EXPEC/UPEC	*aslA*, *astA*, ***chuA***** ^a^**, ***fyuA***** ^a^**, *hha*, *iss*, *lpfA*, *nlpI*, ***papA***** ^b^**, ***papC***** ^b^**, *pic*, *shiB*, *terC*, ***vat***** ^a^**, *yehB*, *yehC*, *yehD*	*cea*, *csgA*, *fdeC*, *irp2*, *mcbA*, *ompT*, *traT*	I-E	8, 23	A	-	*bla*_CTX-M-14_, *bla*_TEM-1B_, *qnrS1*	117/F
**X5723** **(PN)**	4	IncFIB, IncI1, IncQ1, IncX1	MOB_F12_, MOB_P12_, MOB_P12_, MOB_P3_	EXPEC	*aslA*, *capU*, *cvaC*, ***fyuA***** ^a^**, *gad*, *hlyE*, *irp2*, *kpsE*, ***kpsMII***** ^b^**, *nlpI*, *shiA*, *terC*, *yehA*, *yehB*, *yehC*, *yehD*	*anr*, *cea*, *cib*, *cma*, *csgA*, *fdeC*, *hlyF*, *iroN*, *irp2*, *iss*, *iucC*, ***iutA***** ^b^**, *ompT*, *sitA*, *traJ*	I-E	7, 5	C	-	*bla*_CTX-M-1_, *bla*_TEM-1B_, *aadA1*, *aadA2*, *dfrA1*, *lnu*(F), *mef*(C), *mph*(B), *mph*(G), *qacE*, *sul1*, *sul2*, *tet*(A)	93/A
**X3314 (CM)**	4	Col8282, IncB/O/K/Z, IncQ1, p0111	MOB_P12_, MOB_P12_	UPEC	*aslA*, *air*, ***chuA***** ^a^**, *eilA*, *espY2*, *fimH*, ***fyuA***** ^a^**, *gad*, *hha*, *hlyE*, *hra*, *iha*, *ireA*, *irp2*, *kpsE*, ***kpsMII* ^b^**, *neuC*, *nlpI*, *shiB*, *terC*, *yehA*, *yehB*, *yehC*, *yehD*	*colE2-like*, *colE9*, *csgA*, *fdeC*, *irp2*, *iucC*, ***iutA***** ^b^**, *ompT*, *sitA*, *traT*	I-E	10, 22	B	-	Susceptible	115/D
**X4879** **(PN)**	4	IncFIB, IncI1, IncR, IncX1	MOB_F12_, MOB_P12_, MOB_P3_	-	*aslA*, *capU*, *espY2*, *fimH*, *gad*, *hlyA*, *hlyE*, *iss*, *nlpI*, *sepA*, *terC*, *yehA*, *yehB*, *yehC*, *yehD*	*csgA*	I-E, I-F1	11, 25, 4	A *	-	*aph(4)-Ia*, *aac(3)-IV*, *aph(6)-Id*, *aph(3″)-Ib*, *aph(3″)-Ib*, *aph(3″)-Ib*, *aph(3″)-Ib*, *aph(3′)-Ia*, *aadA3*, *aadA24*, *aadA22*, *aadA1*, *aadA1*, *ant(3″)-Ia*, *bla*_TEM-1B_, *erm*(B), *floR*, *cmlA1*, *qnrS1*, *sul2*, *sul1*, *sul3*, *tet*(D), *dfrA12*	1011/A
**X5102** **(PN)**	2	IncR, IncX1	MOB_P3_	-	*aslA*, *fimH*, *gad*, *hlyE*, *iss*, *nlpI*, *terC*, *yehA*, *yehB*, *yehC*, *yehD*	*csgA*, *fdeC*, *ompT*	I-E	4, 9	A *	-	*aadA2*, *bla*_TEM-1B_, *mph*(A), *sul1*, *sul3*, *tet*(A), *dfrA12*	3892/A
**X3071 (CM)**	2	IncFIB-IncFII, IncX1	MOB_F12_, MOB_P3_	-	*aslA*, *air*, *chuA*, *eilA*, *espY2*, *fimH*, *gad*, *hlyE*, *iss*, *nlpI*, *terC*, *yehB*, *yehC*, *yehD*	*cea*, *csgA*, *fdeC*, *ompT*	I-E	7, 13	A *	-	a*adA2*, *aac(3)-IId*, *bla*_TEM-143_, *mph*(A), *sul1*, *tet*(A), *dfrA12*	48/D
**X9848** **(SN)**	3	Col156, IncB/O/K/Z, IncFIB-IncFII	MOB_P12_, MOB_F12_	STEC	*astA*, *ehxA*, *espI*, *fimH*, ***fyuA***** ^a^**, *gad*, *hha*, *hlyE*, *iha*, *ireA*, *kpsE*, *lpfA*, *nlpI*, *shiB*, *subA*, *terC*, *tia*, *yehA*, *yehB*, *yehC*, *yehD*, ***stx1***** ^e^, *stx2* ^e^**	*cea*, *cia*, *csgA*, *fdeC*, *iss*, *ompT*, *traT*	I-E	4, 9	B	-	Susceptible	4058/B1

^a^ BF-a, broiler farm air; BF-m, manure of broiler farm; CM, chicken meat; PF-m, manure of pig farm; SN, cloacal sample of stork nestling; S, stork fecal sample; PN, pig nostril. ^b^ EAEC isolates were classified as presumptive ExPEC if they were positive for ≥2 of the following virulence genes: *papA* and/or *papC* (P fimbriae), *sfa-focDE* (S and F1C fimbriae), *afa-draBC* (Dr-binding adhesins), *iutA* (aerobactin siderophore system), and *kpsMII* (group 2 capsules). ^c^ EAEC strains were classified as presumptive UPEC if they were positive for ≥2 of the following genes: *chuA* (heme uptake), *fyuA* (yersiniabactin siderophore system), *vat* (vacuolating toxin), and *yfcV* (adhesin). ^d^ EPEC strains are identified due to the presence of the *eae* gene. ^e^ ETEC strains are identified by the presence of *eltAB* or *sta* genes. ^f^ STEC strains are identified due to the presence of *stx1* or *stx2* genes. * Indicates a variation of A type CRISPR/Cas structures.

**Table 2:** Genomic diversity and features among the small plasmids found in the *E. coli* collection surveyed. Plasmids are listed according to the strain in which they are harbored (if ≥1 by isolate, they are enumerated).

Small Plasmid (Nº Strain)	Size (bp)	Relaxase	Replicon Type	Accessory Genes (Proteins)	≥99% Identical Plasmids (Strain Host)
**X7761**	6647	-	Col(MG828)	*orf1* (replication protein)	NC008486 (human)
**X7553**	4356	-	Col(MG828)	*orf1*	CP134374 (chicken)
**C10524**	3078	MOB_Q12_	Col(156)	*cea* (colicin E1), *orf1*	JAIWLQ010000445 (red deer)
**X7163**	3850	MOB_Q12_	Col(156)	*orf1*, *mobA/mobL*, *orf2* (DUF1640 domain-containing protein), *orf3* (Cytoplasmatic protein), *traD*	CP128901 (human)
**X7324**	3904	MOB_Q12_	Col(156) (2)	*orf1*, *mobA/mobL*, *orf2, orf3, traD*	-
**X7757**	5032	MOB_Q12_	Col(156)	*orf1*, *traD*	CP128910 (human)
**X9848**	3887	MOB_Q12_	Col(156)	*orf1*, *mobA/mobL*, *orf1*, *orf2*, *traD*	CP128886 (human)
**X7324, S**	3457	MOB_Q12_	Col(156) (2)	*orf1*, *mobA/mobL*, *orf2*, *orf3*, *traD*	CP128985 (human)
**X7604-1**	6123	MOB_Q12_	Col(156)	*orf1*, *mobA/mobL*, *orf2*, *orf3*, *traD*	AP017615 (wild boar)
**X9848**	6234	MOB_Q12_	Col(156)	o*rf1*, *mobA/mobL*, *traD*, *col* (colicin 8), *cei* (colicin immunity), *cel* (colicin lysis)	CP128895 (human)
**X2583-1**	4077	MOB_V2_	Col(8282)	*orf1*, *imm* (Colicin-E9 immunity protein), *cea* (colicin E1), *exc2* (entry exclusion protein 2), *exc1* (entry exclusion protein 1), *mbeA* (Mobilization protein), *mbeC* (Mobilization protein), *rop*	-
**X3314**		MOB_V2_	Col(8282)	*orf1*, *orf2*, *orf3* (hypothetical)	CP128962 (human)
**X2583-2**		-	Col(pVC)	*orf1*, *exc2*, *exc1*, *cea*, *imm*, *mbeB*, *mbeC*, *mbeD*, *orf2*, *rop*	JX133088 (poultry carcasses)
**X3078**	2376	-	Col(pVC)	orf1 (replication protein)	-
**X3561**	1784	-	Col(pVC)	*orf1*, *exc2*, *exc1*, *cea*, *imm*, *mbeB*, *mbeC*, *mbeD* (Mobilization protein *MbeD*), *orf2*, *rop*	JAIWLI010000409 (red deer)
**X5279**	8899	-	Col(pVC)	*exc2*, *exc1*, *cea*, *imm*, *mbeB*, *mbeC*	-
**X7604-2**	1978	-	Col(pVC)	*orf1*, *exc2*, *exc1*, *cea*, *imm*, *mbeD*	-
**X5726**	6743	MOB_QU_	Col(E10)	*orf1*, *mobQ*, *colE9* (colicin E9), *imm*, *hic* (lysis protein for colicin E2 and E3)	CP075726 (pig)

## Data Availability

The data that support the findings of this study are available in the Supplementary Materials of this article.

## Supplementary Materials

The following supporting information can be downloaded at: https://www.mdpi.com/article/10.3390/pathogens15080812/s1, Table S1. Genomic features of the 38 *Escherichia coli* genomes analysed. Table S2. Information about the genome sequences downloaded from the NCBI for the construction of the phylogenomic core tree. Table S3. Pairwise allelic differences distance matrix calculated from the core genome in the set of 200 genomes recovered from Enterobase. Figure S1. Statistical analysis of *Escherichia coli* isolates using the Mann–Whitney U test: (a) adherence/invasion levels strain by strain, (b,c) adherence/invasion levels grouped by enzyme/pathotype and vice versa, (d,e) adherence/invasion levels grouped by genetic lineage/pathotype and vice versa, (f) biofilm formation strain by strain. Figure S2. Phylogenetic UPGMA tree constructed according to the nucleotide sequences of 32 putative *iss* genes from our *Escherichia coli* collection. Figure S3. Phylogenetic relationship of the 38 studied *E. coli* and 200 reference genomes (Table S2). The tree was based on a 575,173.5 ± 3,802.95 bp core genome including 674 orthol ogous common genes, from a total of 108,518 gene clusters, with at least 80% identity and 90% coverage and using 100 bootstrapping replicates. ST types are highlighted in different colors and origin has been indicated in the right part. The pairwise SNP distance matrix used to build the tree is shown in Table S3. Figure S4. Structural complexity of the CRISPR/Cas I-E and I-F1 systems in terms of the module enclosed by the two CRISPR arrays, encompassing the *cas* set of neighboring genes and ORFs are listed by A, A* (absence of a complete copy of *cse2*), B or C. The CRISPR arrays illustrated in this figure depict only their position within the CRISPR/Cas systems (the spacer repertoire of each isolate is presented in Figure 1). The *cas*-E genes (*cas2*, *cas1*, *cas6*, *cas5*, *cas7*, *cse2*, *cse1* and *cas3*) in pink and *cas*-F1 genes (*cas1-cas2/3-csy1-csy2-csy3-cas6f*) in turquoise, are co-located between the CRISPR-1/CRISPR-2 loci in the IE system and CRISPR-3/CRISPR 4 in the I-F1 system. Figure S5. The UPGMA phylogenetic tree created using the combined nucleotide sequences of the *cas* genes. Different phylogenetic groups are indicated by coloured circles (red: A; dark blue: B1; pink: B2; brown: C; green: D; light blue: E; yellow: G). Additionally, the concatenated *cas*-E genes from *E. coli* K-12 MG1655 serve as a reference.
