# Supplementary material for: Virulence and Invasion Profiles of Escherichia coli Across One Health Reservoirs: Genomic Insights into High-Risk Clones and Their Defense Systems
Source: Pathogens. 2026 Aug 1;15(8):812. doi: 10.3390/pathogens15080812 (PMC13516913; doi:10.3390/pathogens15080812)
Supplement: Supplementary file 1 [file pathogens-15-00812-s001.zip › Supplementary_figures_pathogens.pdf]

(a)

ADHERENCE

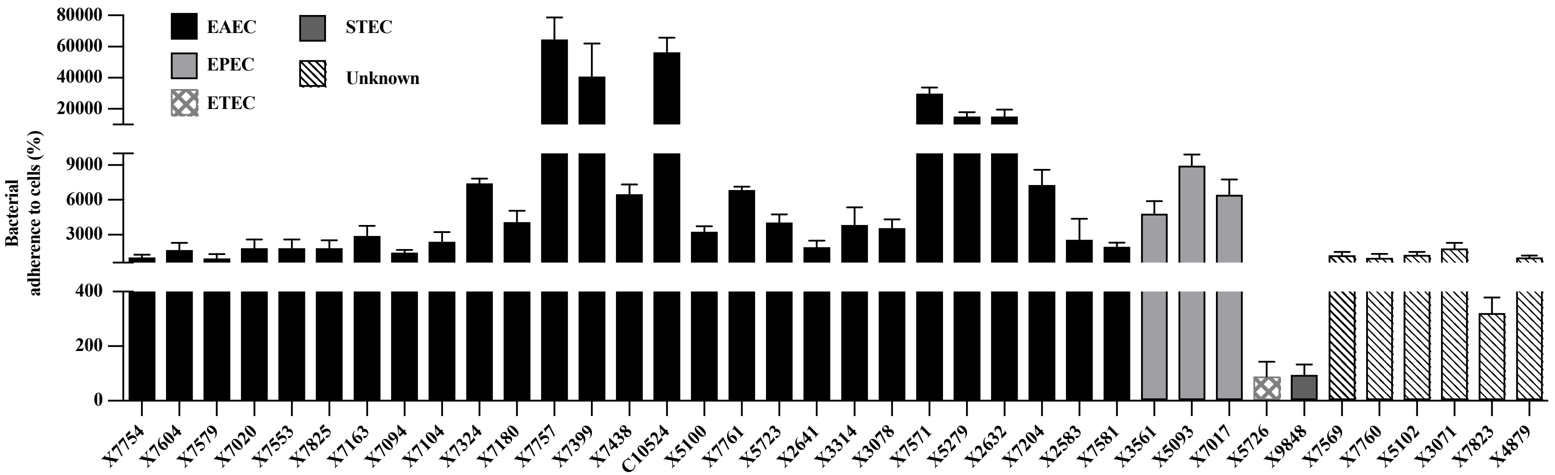

INVASION

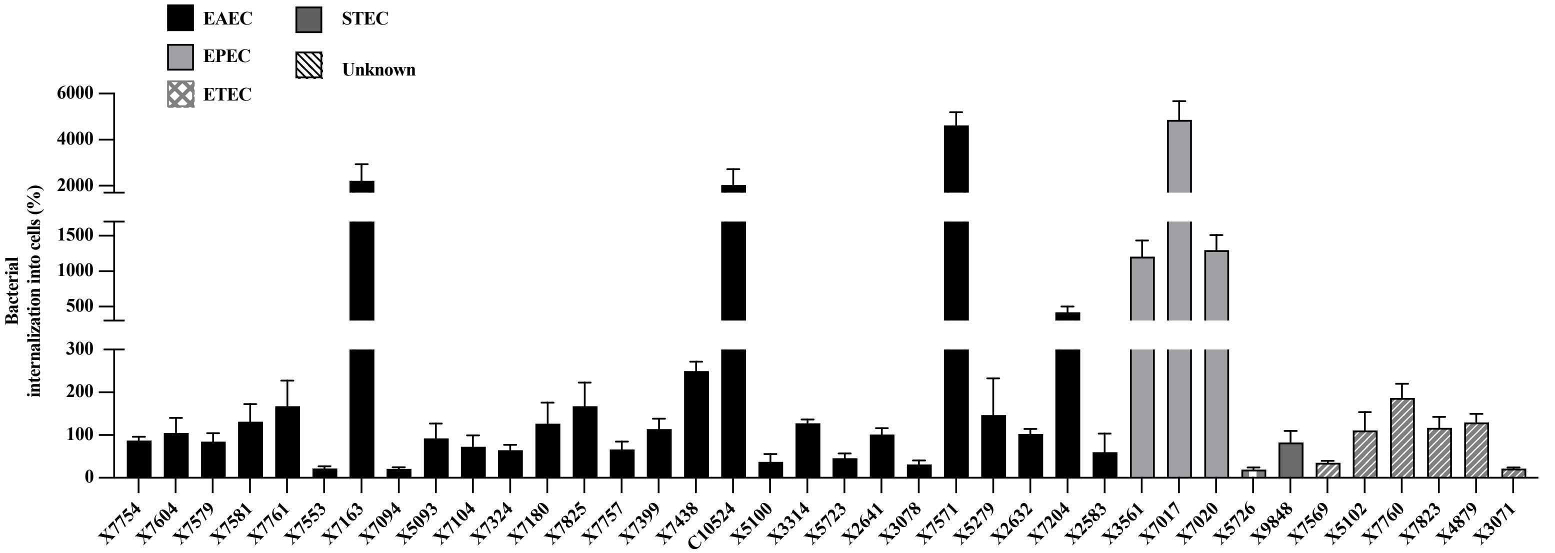

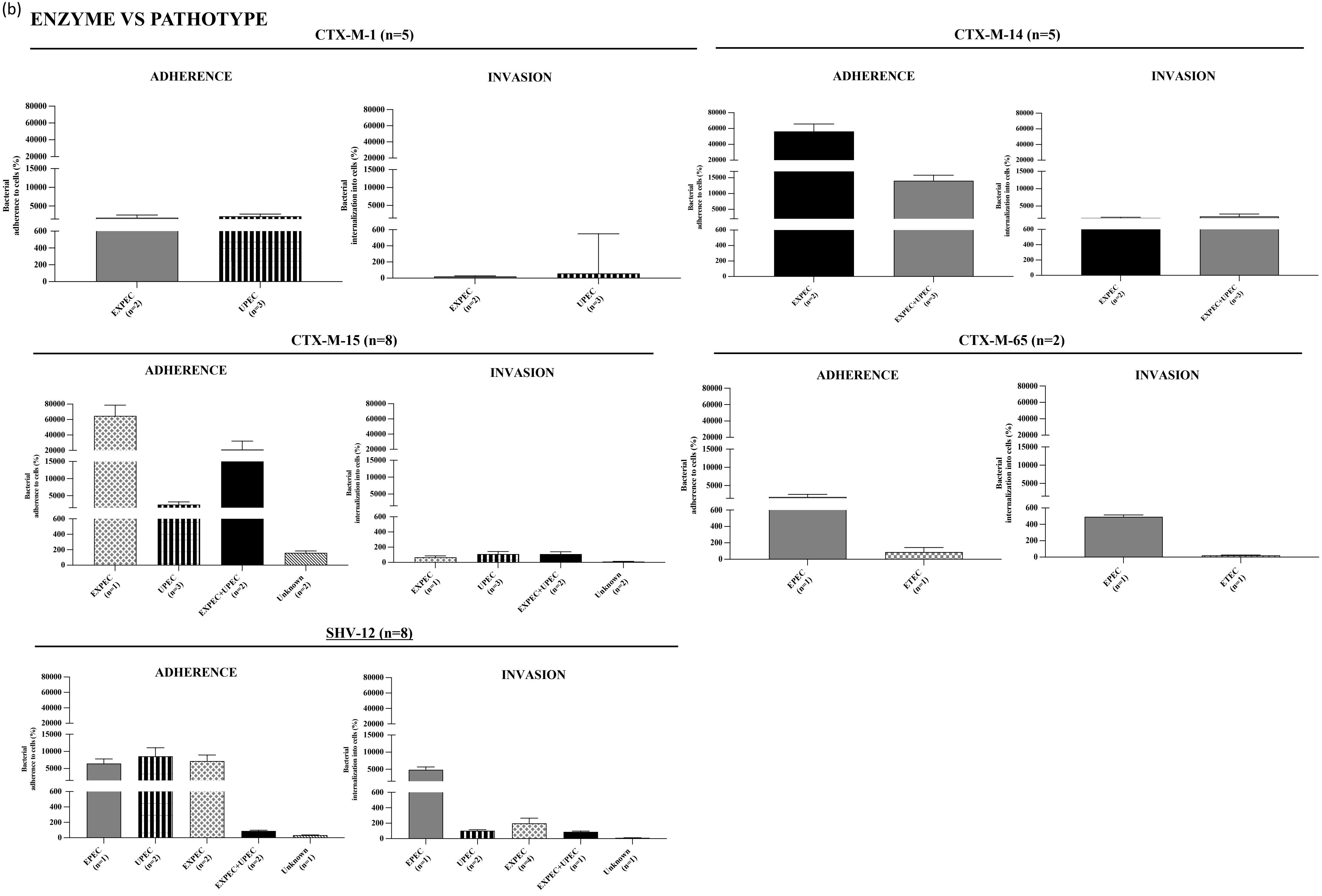
(b)
ENZYME VS PATHOTYPE
CTX-M-1 (n=5)
ADHERENCE
INVASION
Bacterial adherence to cells (%)
Bacterial internalization into cells (%)
EXPEC (n=2)
UPEC (n=3)
CTX-M-14 (n=5)
EXPEC+UPEC (n=3)
CTX-M-15 (n=8)
EXPEC (n=1)
EXPEC+UPEC (n=2)
Unknown (n=2)
CTX-M-65 (n=2)
EPEC (n=1)
ETEC (n=1)
SHV-12 (n=8)
UPEC (n=2)
EXPEC (n=2)
Unknown (n=1)
EXPEC (n=4)
EXPEC+UPEC (n=1)
0
200
400
600
5000
10000
15000
20000
40000
60000
80000

(c)

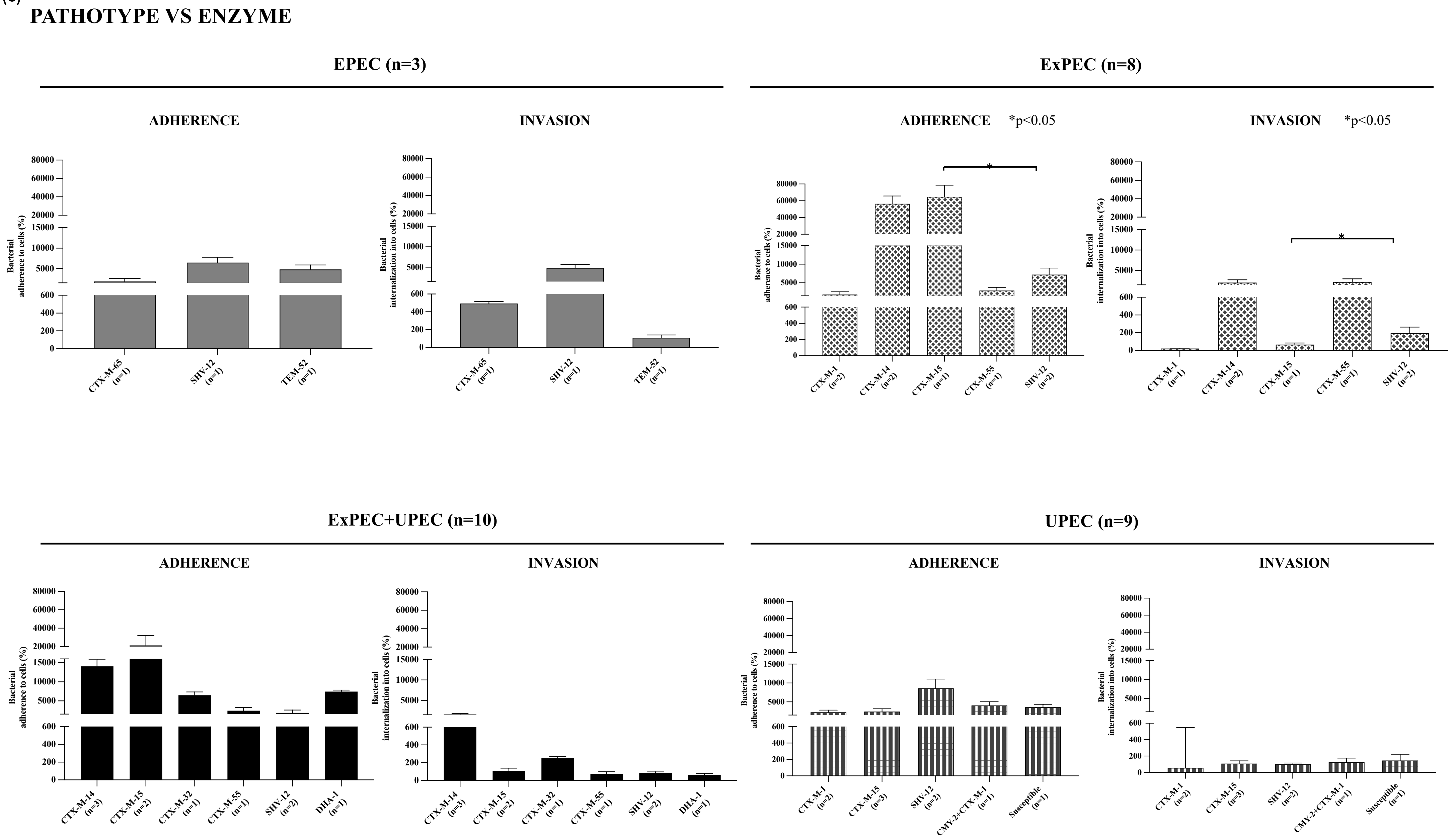
PATHOTYPE VS ENZYME
EPEC (n=3)
ADHERENCE
INVASION
Bacterial adherence to cells (%)
Bacterial internalization into cells (%)
CTX-M-65 (n=1)
SHV-12 (n=1)
TEM-52 (n=1)
ExPEC (n=8)
ADHERENCE *p<0.05
INVASION *p<0.05
CTX-M-1 (n=2)
CTX-M-14 (n=2)
CTX-M-15 (n=1)
CTX-M-55 (n=1)
SHV-12 (n=2)
ExPEC+UPEC (n=10)
CTX-M-14 (n=3)
CTX-M-15 (n=2)
CTX-M-32 (n=1)
CTX-M-55 (n=1)
SHV-12 (n=2)
DHA-1 (n=1)
UPEC (n=9)
CTX-M-1 (n=2)
CTX-M-15 (n=3)
SHV-12 (n=2)
CMY-2+CTX-M-1 (n=1)
Susceptible (n=1)

(d)

PATHOTYPE VS GENETIC LINEAGE

EPEC (n=3)

ADHERENCE

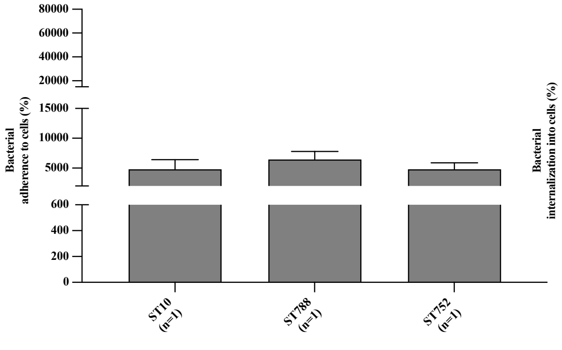

INVASION

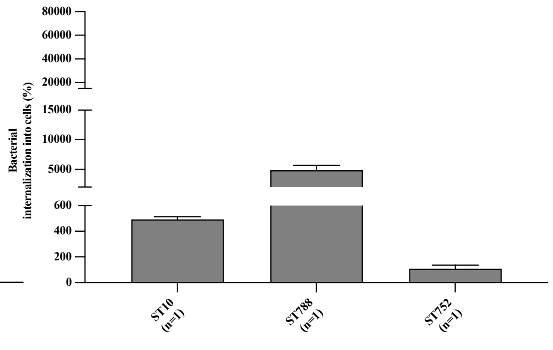

ExPEC (n=8)

ADHERENCE

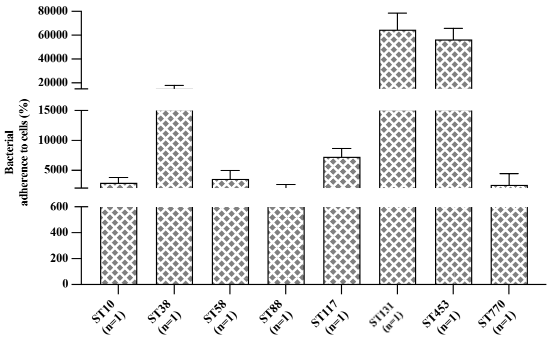

INVASION

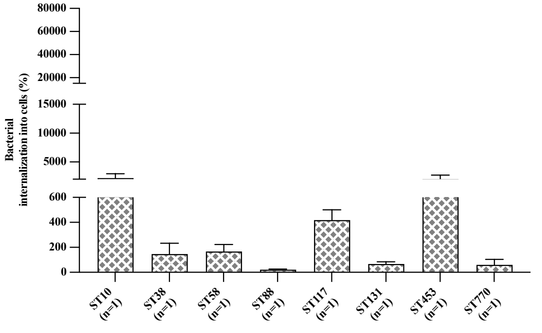

ExPEC+UPEC (n=10)

ADHERENCE

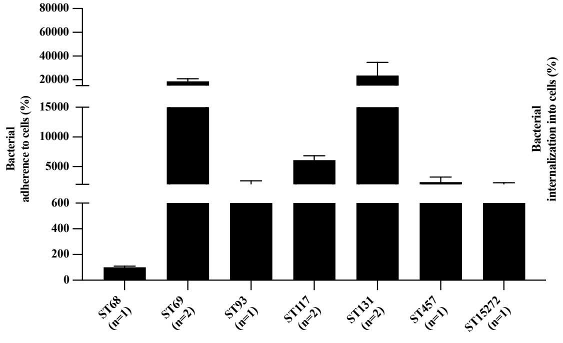

INVASION *p<0.05

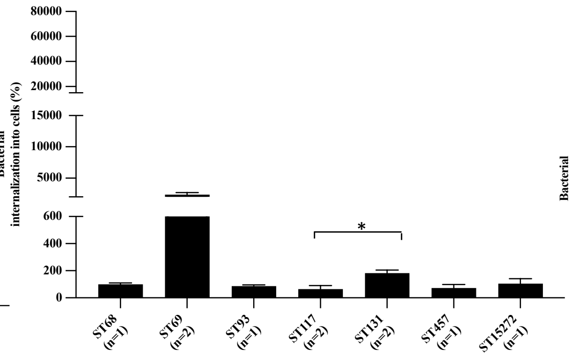

UPEC (n=9)

ADHERENCE

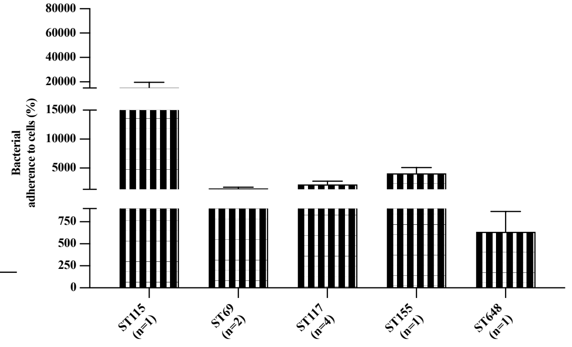

INVASION *p<0.05

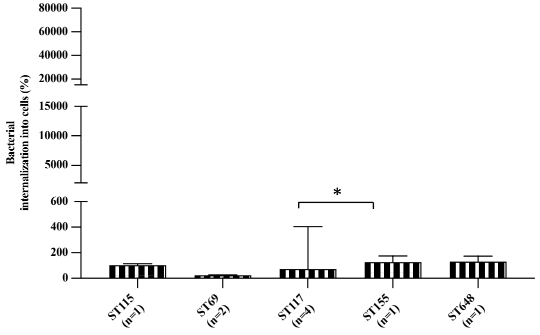

(e) **PATHOTYPE VS GENETIC LINEAGE**

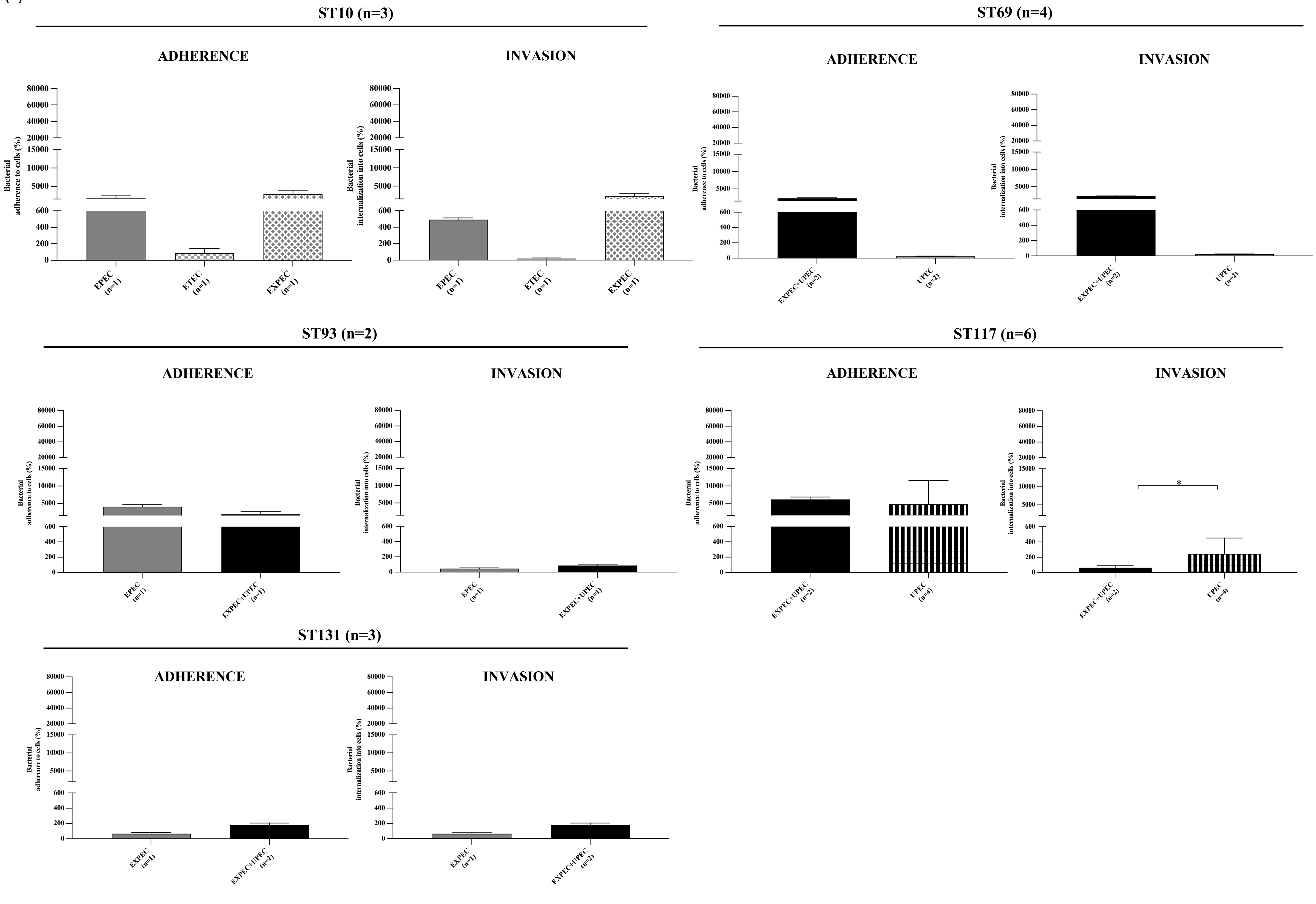

(f) **BIOFILM FORMATION**

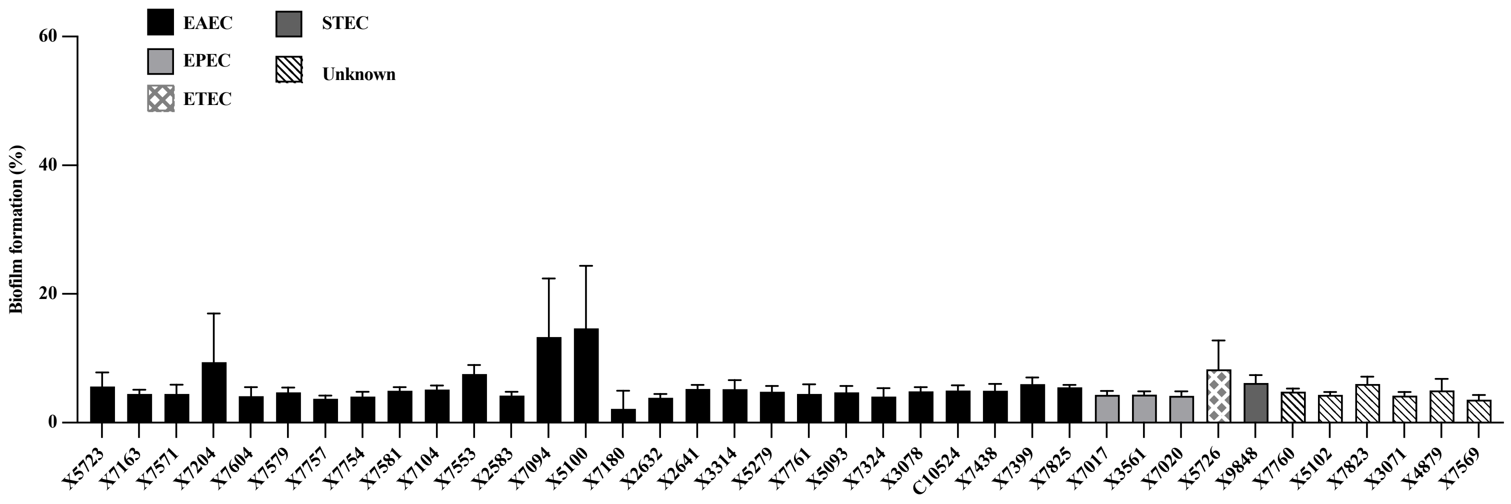

Figure S1. Statistical Analysis of *Escherichia coli* isolates Using the Mann-Whitney U Test: (a) adherence/Invasion levels strain by strain, (b/c) adherence/invasion levels grouped by enzyme/pathotype and viceversa, (d/e) Adherence/invasion levels grouped by genetic lineage/pathotype and viceversa, (f) Biofilm formation strain by strain.

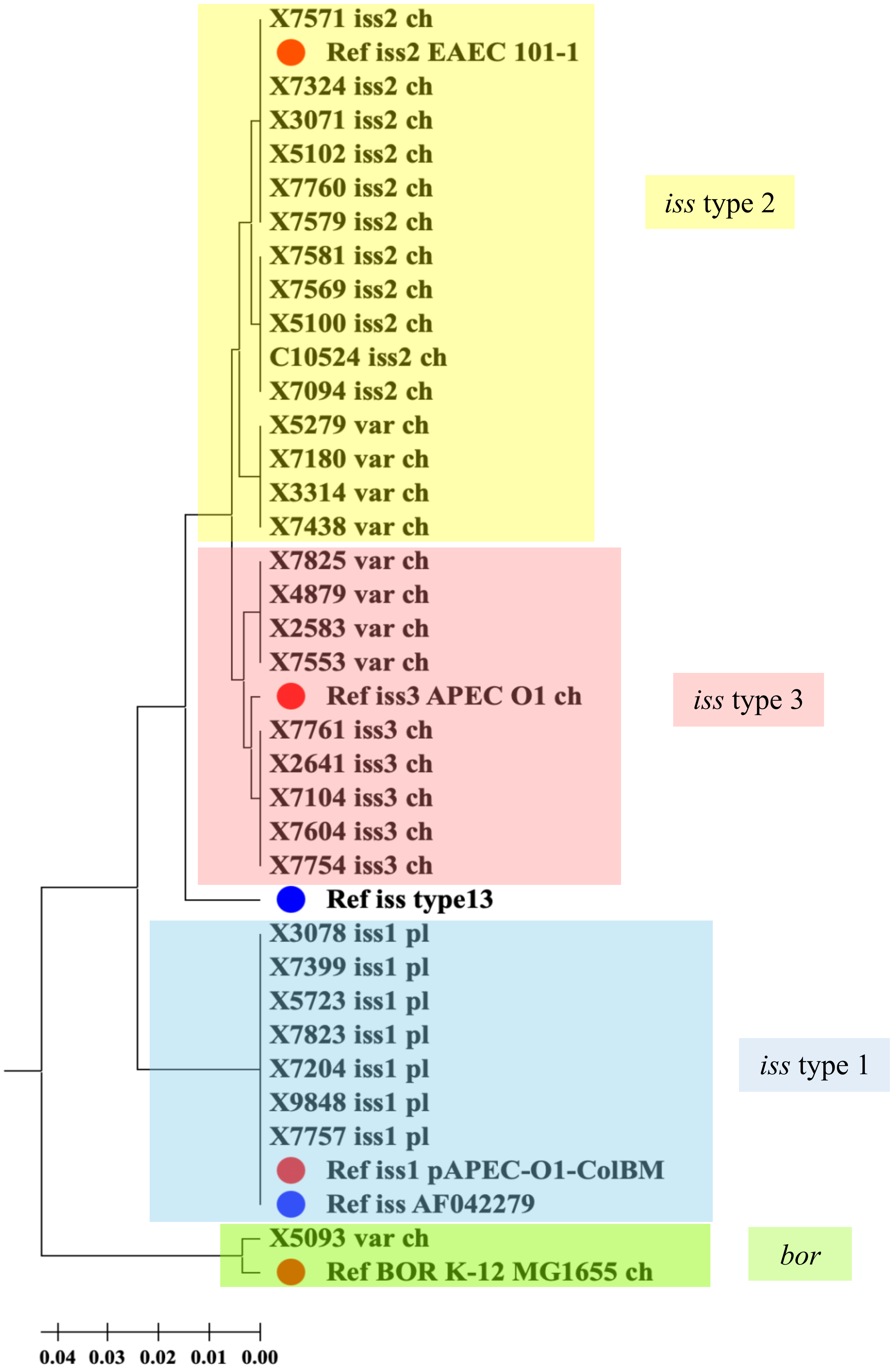

Figure S2. Phylogenetic UPGMA tree constructed according to the nucleotide sequences of 32 putative *iss* genes from our *E. coli* collection. Some reference sequences were used by Johnson et al. 2008 and Xy et al. 2018 in order to refer to high (AF042279) or low (*iss* type 13) serum livability of the *iss* gene (blue dots). The distinct variants of *iss* gene are included and marked with red dots

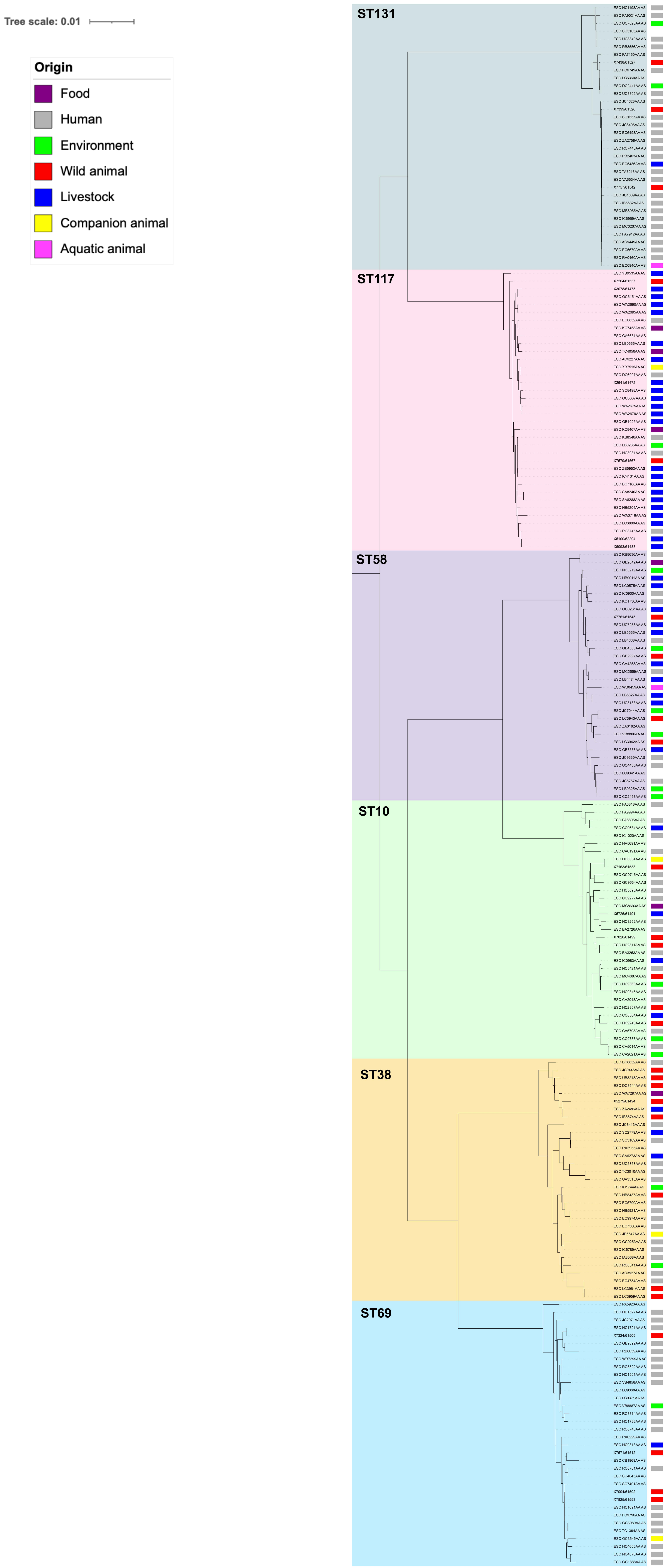

Figure S3. Phylogenetic relationship of the 38 studied *E. coli* and 200 reference genomes (Table S2). The tree was based on a 575,173.5 ± 3,802.95 bp core genome including 674 orthol ogous common genes, from a total of 108,518 gene clusters, with at least 80% identity and 90% coverage and using 100 bootstrapping replicates. ST types are highlighted in different colors and origin has been indicated in the right part. The pairwise SNP distance matrix used to build the tree is shown in Table S3.

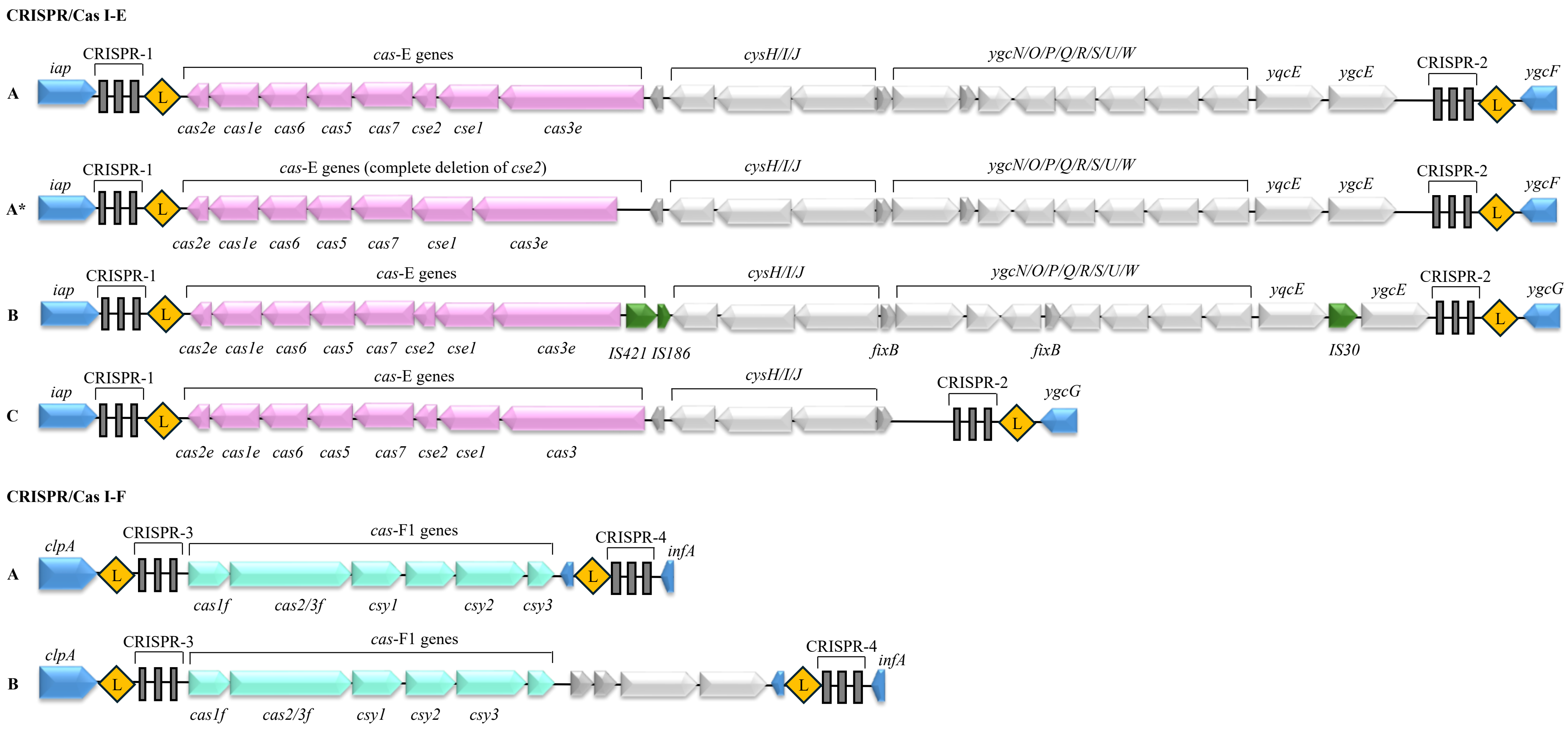

Figure S4. Structural complexity of the CRISPR/Cas I-E and I-F1 systems in terms of the module enclosed by the two CRISPR arrays, encompassing the *cas* set of neighboring genes and ORFs are listed by A, A*(absence of a complete copy of *cse2*), B or C. The CRISPR arrays illustrated in this figure depict only their position within the CRISPR/Cas systems (the spacer repertoire of each isolate is presented in Figure 1). The *cas*-E genes (*cas2, cas1, cas6, cas5, cas7, cse2, cse1* and *cas3*) in pink and *cas*-F1 genes (*cas1-cas2/3-csy1-csy2-csy3-cas6f*) in turquoise, are co-located between the CRISPR-1/CRISPR-2 loci in the I-E system and CRISPR-3/CRISPR 4 in the I-F1 system.

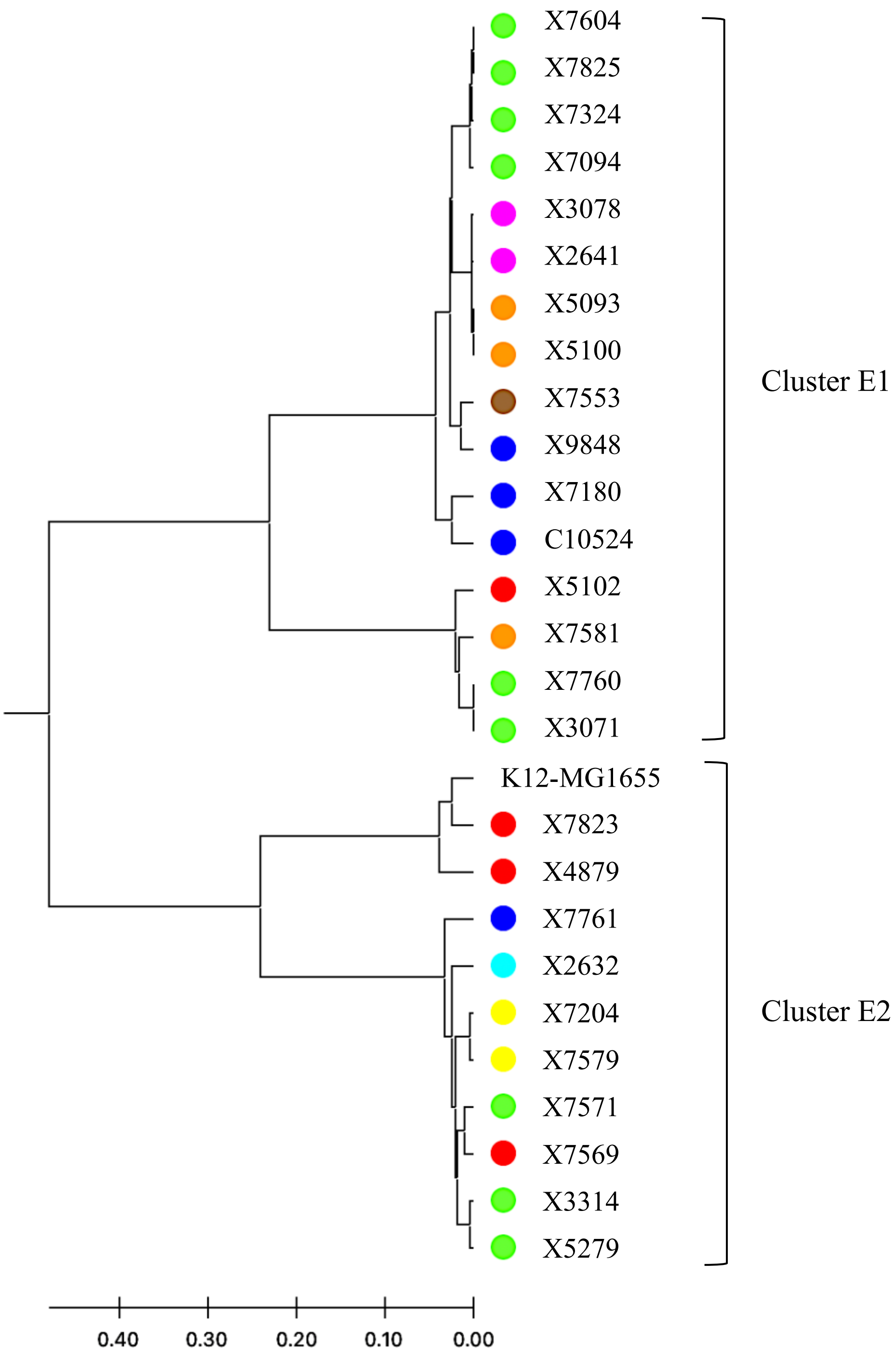

Figure S5. The UPGMA phylogenetic tree was created using the combined nucleotide sequences of the *cas* genes. Different phylogenetic groups are indicated by coloured circles (red: A; dark blue: B1; pink: B2; brown: C; green: D; light blue: E; yellow: G). Additionally, the concatenated *cas-E* genes from *E. coli* K-12 MG1655 serve as a reference.
